# Assisted delivery of anti-tumour platinum drugs using DNA-coiling gold nanoparticles bearing lumophores and intercalators: towards a new generation of multimodal nanocarriers with enhanced action†
†Electronic supplementary information (ESI) available: Additional characterisation, DNA binding and TEM cell uptake data. See DOI: 10.1039/c9sc02640a


**DOI:** 10.1039/c9sc02640a

**Published:** 2019-08-13

**Authors:** Ana B. Caballero, Lucia Cardo, Sunil Claire, James S. Craig, Nikolas J. Hodges, Anton Vladyka, Tim Albrecht, Luke A. Rochford, Zoe Pikramenou, Michael J. Hannon

**Affiliations:** a School of Chemistry , University of Birmingham , Edgbaston , Birmingham B15 2TT , UK . Email: m.j.hannon@bham.ac.uk ; Email: z.pikramenou@bham.ac.uk; b Physical Sciences for Health Centre , University of Birmingham , Edgbaston , Birmingham B15 2TT , UK; c School of Biosciences , University of Birmingham , Edgbaston , Birmingham B15 2TT , UK

## Abstract

New gold and lipoic based nanocarriers for the delivery of platinum(ii) and platinum(iv) drugs are developed, which allow enhanced loading of the drug on the surface of the nanocarriers and release in a pH-dependent fashion, with superior release at lower pHs which are associated with many tumours. The conjugate nanoparticles and their conjugates enter cells rapidly (within 3 hours). They tend to cluster in vesicles and are also observed by light and electron microscopies in the cytoplasm, endoplasmic reticulum and nucleus. We further incorporate aminoanthraquinone units that are both fluorophores and DNA intercalators. This results in nanocarriers that after drug release will remain surface decorated with DNA-binders challenging the conventional design of the nanocarrier as an inert component. The outcome is nanocarriers that themselves have distinctive, remarkable and unusual DNA binding properties being able to bind and wrap DNA (despite their anionic charge) and provide enhanced cytotoxic activity beyond that conferred by the platinum agents they release. DNA coiling is usually associated with polycations which can disrupt cell membranes; anionic nanoparticles that can cause novel and dramatic effects on DNA may have fascinating potential for new approaches to in-cell nucleic acid recognition. Our findings have implications for the understanding and interpretation of the biological activities of nanoparticles used to deliver other DNA-binding drugs including clinical drug doxorubicin and its formulations.

## Abstract 2

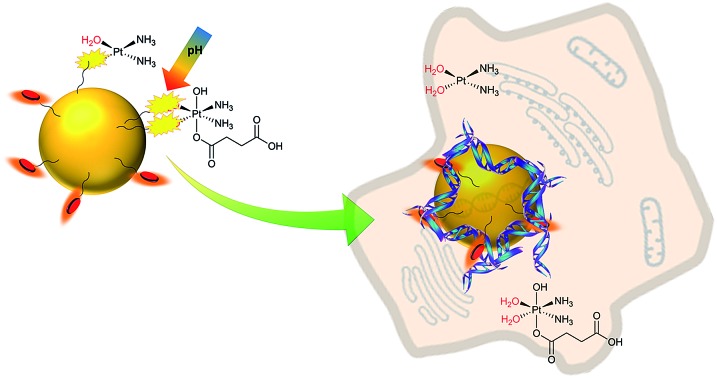
Nanocarriers with unusual DNA binding properties provide enhanced cytotoxic activity beyond that conferred by the platinum agents they release.

## Introduction

The use of nanocarriers has attracted much recent interest for improving delivery of drugs toward specific biological targets to achieve safer and more effective therapy.^1^–^5^ The conjugation of a drug to a nanovehicle might also prevent its deactivation by other biomolecules and enhance its bioavailability and stability. Smart designs can be further employed to control drug release kinetics by internal or external stimuli and bypass standard multidrug resistance mechanisms by opening up alternative uptake processes.^1^,^6^–^11^

Cisplatin, and five other similar designs of platinum compounds, are crucial clinical drugs for many cancer treatments, with cisplatin, carboplatin and oxaliplatin licensed worldwide and used to treat many patients each year.^12^–^16^ Despite their great efficacy, the use of platinum drugs is limited by severe toxic effects, problems in bioavailability and acquired resistance, among other factors. Toxicity of current platinum drugs is in part due to their lack of specificity towards cancer cells. This is aggravated by relatively high doses of drug needed to be effective, since these compounds often suffer inactivation by diverse biomolecules present in the organism, such as glutathione and metallothionein, and only a tiny fraction reaches their target, DNA.^17^,^18^

As such, platinum drugs are strong candidates for nanocarrier delivery and a variety of different types of nanocarriers have been explored: many of them have made significant progress in terms of solubility, cell uptake and efficacy and some nano-formulations have entered clinical trials, but none have yet been approved.^5^,^19^–^24^

Gold nanoparticles (GNPs) have been shown to be versatile drug-delivery platforms for different drugs, allowing easy conjugation procedures and the possibility of co-loading different functionalizing units, such as targeting vectors or imaging probes to modify and control biodistribution.^4^,^25^–^29^ In addition, GNPs are themselves biocompatible, non-toxic and inert; their size and dispersity is easy to control and small GNPs efficiently penetrate cell membranes and can be monitored inside cells using a variety of modalities.^30^–^32^ Anionic nanoparticles are perceived to be advantageous for drug delivery, as cationic nanoparticles can disrupt membranes in the cell.^33^,^34^

A few studies exploring the potential of GNP-assisted delivery of platinum drugs have been reported: carboxylate-functionalized GNPs have been synthesized to conjugate cisplatin^35^–^40^ and oxaliplatin^41^ by direct binding of Pt(ii) to carboxylic groups; cisplatin has also been attached to GNPs by interaction with PEG chains^42^ and lipidic coatings forming two or three layers;^43^ Pt(iv) prodrugs have been attached to GNPs surface either by amide bonds through their labile axial ligands,^44^ and non-covalently onto a GNP surface through cyclodextrins (guest–host chemistry).^45^ Some of these examples have employed release triggering mechanisms using pH-sensitive carboxylate linkages^39^,^40^,^46^ and thiol groups.^42^

The current paradigm is that the nanocarrier is normally designed as an inert component, with a role just to deliver and release the drug. Yet the potential of these nanosystems is much greater. Herein we challenge this paradigm through design of a novel GNP-based nanocarrier that not only enables high drug loading and subsequent release of platinum drugs in cells (thereby enhancing their activity), but that also bears organic units that are both fluorophores and DNA intercalators. The result is nanocarriers that are not inert but themselves have remarkable DNA binding properties being able to bind and wrap DNA, providing enhanced cytotoxic activity beyond that conferred by the platinum agents they release.

## Results and discussion

### Molecular design

**Fig. 1:**
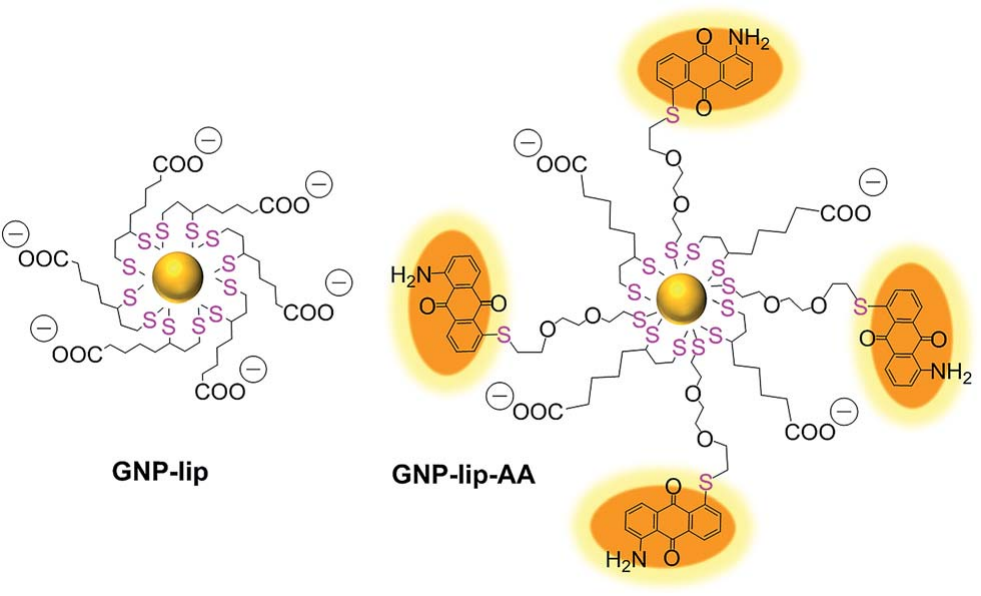
GNP-lip and fluorescently labelled GNP-lip-AA nanocarriers for platinum drug delivery.

We designed a GNP-based platform, the two systems reported in Fig. 1, GNP-lip and GNP-lip-AA, both functionalized with α-lipoic acid (a molecule produced and present naturally in the body) as coating material able to provide high stability and biocompatibility to the carriers;^47^ the anionic lipoic unit is a shorter linker compared to other carboxylic acid based linkers employed in previous works,^35^–^41^ but still able to form pH-sensitive bonds with platinum drugs and stabilize the systems enough to allow post-functionalization with positively charged moieties. GNP-lip-AA introduces the DNA-intercalating fluorescent aminoanthraquinone (AA) anchored to the nanoparticle as second functionality. AA was anchored to GNP by a thiolated ethylene glycol linker, whose length was selected to avoid steric hindrance effects that might affect the DNA-intercalation properties.

GNP-lip and GNP-lip-AA were then employed as carriers of both cisplatin (PtII) and the Pt(iv) prodrug, *c*,*c*,*t*-[Pt(NH_3_)_2_Cl_2_(OH)(Hsuc)] (PtIVsuc), where Hsuc is the monohydrogensuccinate anion.^44^ A feature of the design approach is that the platinum centres are coordinated directly to the lipoic surface of the nanoparticle, rather than using a pre-formed Pt drug-linker system to attach to the gold below the surface layer.

### Nanocarrier synthesis and characterization

The GNP-lip carrier was synthesized by coating citrate gold nanoparticles with α-lipoic acid. GNP-lip-AA was then prepared by adding the AA ligand (where the aminoanthraquinone is previously functionalized with the ethylene glycol linker) to the GNP-lip system (Fig. 1, S1 and S2†). Due to the negatively-charged surface of both carriers, all functionalization steps were carried out in basic media (pH > 10). Pre-coating of GNP with anionic lipoate (to form GNP-lip) is essential to prevent particle aggregation as observed when we attempted to functionalize citrate GNP directly with AA.

Because of the ‘double thiol anchoring’ of lipoic acid to GNPs, the displacement of this ligand is slower compared to mono-thiolated ligands and a high excess of the secondary ligand AA and long reaction times (18 h) were required to form stable mixed-ligand particles GNP-lip-AA.

Binding of AA to GNP-lip was confirmed by a red shift in the surface plasmon resonance (SPR) band in the UV-vis spectrum (Fig. S2†). FT-IR spectra also confirmed the presence of both lipoate and AA ligands (from their fingerprints) with the loss of the S–H bond stretching band of both ligands, confirming attachment to gold surface *via* this group (Fig. S3†).

**Scheme 1:**
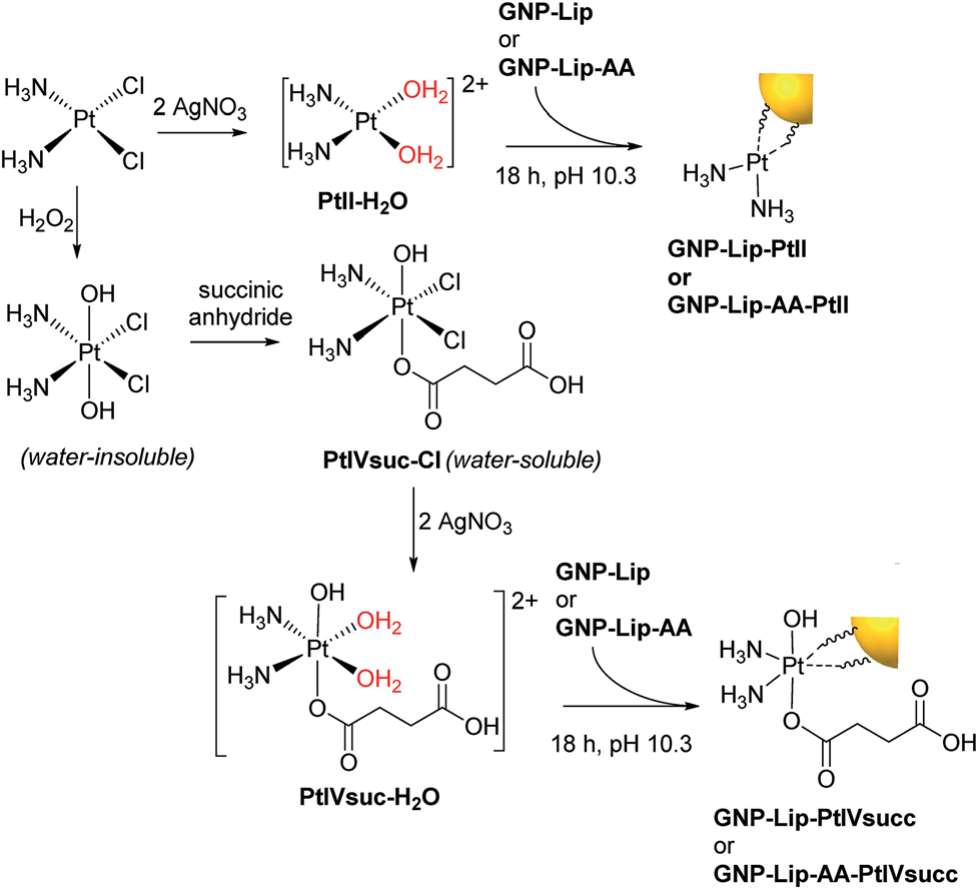
Activation of platinum drugs and attachment onto the GNP surface *via* the carboxylate group of lipoic acid linkers.

To be suitable for conjugation to the carboxylate group of the lipoic linker, the chloride ligands of both cisplatin and Pt(iv) prodrug *c*,*c*,*t*-[Pt(NH_3_)_2_Cl_2_(OH)(O_2_CCH_2_CH_2_CO_2_H)], were removed by precipitation with Ag(i), achieving the activated aquo analogues (Scheme 1). Again, because GNP-lip and GNP-lip-AA are negatively-charged systems, the attachment of platinum drugs was carried out in basic conditions (pH 10), where the rate of platinum binding to GNPs would be controlled by an equilibrium with the formation of less-labile hydroxyl-complexes;^12^ this facilitates a homogeneous binding process while avoiding particle collapse by a total charge neutralization. The S of the lipoic acid is (strongly) bound to the Au of the nanoparticle and the exterior of the nanoparticle presents a surface array of carboxylates as evidenced by the zeta potential.

Characterization of the synthesized GNP conjugates was performed by TEM, DLS, UV-vis and fluorescence studies, and in part summarized in Table 1 (see also S4–S8); the cores of both the GNP-lip and GNP-lip-AA-based systems are *ca.* 14 nm in size with low polydispersity (see also Fig. S4†). The red shift in the UV-vis band (Fig. S5†) is indicative of the change of the surface of the nanoparticle indicating the attachment of the platinum complex.

**Table 1:** Characterization of the nanocarriers before and after attaching PtII and PtIVsuc complexes onto their surface

	GNP-lip	GNP-lip-PtII	GNP-lip-PtIVsuc
Hydrodynamic diameter (nm; from DLS)	10 ± 4	15 ± 5	15 ± 5
Core diameter (nm; from TEM)	14 ± 3	14 ± 3	15 ± 3
*ζ*-potential (mV)	–44 ± 19	–39 ± 6	–43 ± 8
SPR band (nm)	522	525	525

	GNP-lip-AA	GNP-lip-AA-PtII	GNP-lip-AA-PtIVsuc
Hydrodynamic diameter (nm; from DLS)	23 ± 7	25 ± 7	25 ± 7
Core diameter (nm; from TEM)	14 ± 3	15 ± 4	15 ± 4
*ζ*-potential (mV)	–38 ± 6	–34 ± 6	–37 ± 11
SPR band (nm)	528	530	530

All GNP-lip-AA particles, with and without attached platinum drugs, showed analogous emission bands centred at 645 nm upon excitation at 470 nm (Fig. S8†) in water, attributed to the presence of aminoanthraquinone.^48^

**Fig. 2:**
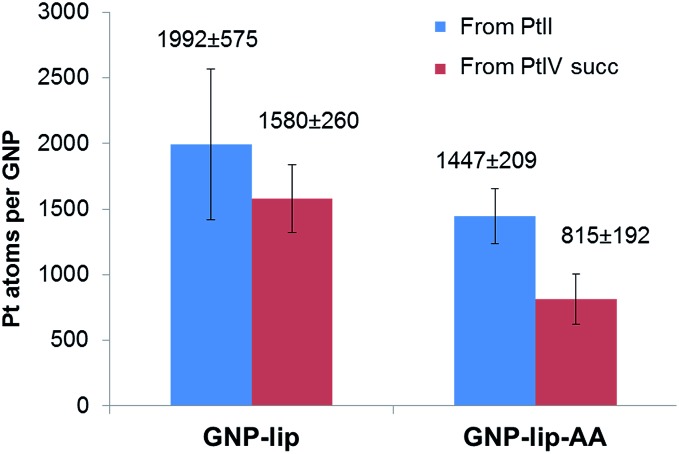
Loading of Pt complexes onto GNP-lip and GNP-lip-AA, measured by ICP-MS analysis. Results are the average of at least four replicates (±SD).

The average drug loading onto GNP-lip and GNP-lip-AA conjugates was determined by calculating the number of Pt atoms per GNP, obtained by measuring the concentration of Pt and Au by ICP-MS (Fig. 2). The amount of platinum per GNP, detected in each conjugated system, reflects the platinum drug/GNP molar ratio employed for their syntheses (see Experimental details), and the loading of Pt(ii)-drug is higher compared to Pt(iv)-drug, consistent with the different reactivity of the two oxidation states. The GNP-lip-AA particles are expected to bear less lipoic sites and the loading is consequently lower. Nevertheless, in all cases, this synthetic strategy, based on pre-coating the GNP with the coordinating linker (lipoate) first, followed by the anchoring of the Pt-drug, yielded significantly higher loading of the drug, than other reported GNP conjugates, in which a pre-formed Pt drug-linker system is attached to particle.¶
¶For example ref. 38 reports 815 Pt(ii) on larger 25 nm GNP, with 3–4 times greater surface area; ref. 41 achieves 280 Pt(ii) on 31 nm GNP; ref. 37 has 1300 Pt(ii) on 25 nm GNP; even with carboxylate dendrimers, ref. 36 achieves just 1000 Pt(ii) per GNP. Probably, nanoparticle pre-coated with the linker presents a higher density of charged anchoring sites on the surface, which helps improving both particle stability and final drug distribution.

### *In vitro* stability

Since these Pt-drug-nanocarrier conjugates were developed to explore their activity in cells, their stability in cell culture media was investigated. Purified suspensions of Pt-containing and non-Pt containing conjugates were centrifuged at 5000*g* for 30 min and the pellets further dispersed in serum-supplemented cell media (RPMI 1640 and DMEM).

UV-Vis spectra and hydrodynamic size determinations were collected at different times (Fig. S9–S10†), showing only minor particle aggregation in cell media over 72 hours. Overall, all colloidal suspension remained stable over the time monitored. According to Liu *et al.*,^48^ certain levels of aggregation would be an advantage for our purposes: nanoparticles with a diameter between 30 and 200 nm can accumulate more effectively inside tumour tissues and therefore retention and cellular uptake of small nanoparticles (<30 nm) can be enhanced by stimulated aggregation of nanoparticles. This also aids their visualization by confocal microscopy, which was used to assess their cell uptake (below).

## *In vitro* drug release

We investigated drug release profiles of the four Pt-containing conjugates (GNP-lip-PtII, GNP-lip-PtIVsuc, GNP-lip-AA-PtII and GNP-lip-AA-PtIVsuc) in different conditions by measuring concentrations of Pt and Au with ICP-MS.

First, we analysed drug release at different times, in cell media (RPMI and DMEM), in an incubator at 37 °C (same conditions employed for *in cellulo* studies, described below). The highest percentage of release occurs within the first 5 hours of incubation: 20% of PtII drug is released from GNP-lip and 40–50% from GNP-lip-AA, while for the analogous PtIVsuc conjugates, ∼50% of the platinum is released from both nanosystems (Fig. S11†).

It should be noted that cell media contains several Pt-binding species at relatively high concentrations, such as l-lysine (40 and 146 mg L^–1^ in RPMI and DMEM), l-cystine (50 and 63 mg L^–1^ in RPMI and DMEM), or reduced glutathione (1 mg L^–1^ in RPMI); an equilibrium between different Pt-containing systems can be reached during the first 5 h (ref. 49) and these would obviously affect Pt-drug release profile.

According with our initial hypothesis, the designed nanocarriers GNP-lip and GNP-lip-AA should also be able to release the platinum drugs in a pH-controlled fashion; therefore, we determined and compared the percentage of drug that is released at (a) pH 7.4 (in phosphate buffered saline, mimicking cellular pH) and (b) pH 4.4 (in phthalate–NaOH buffer, mimicking endosomal environment), which are higher and lower than carboxylic acid p*K*_a_, respectively.

**Fig. 3:**
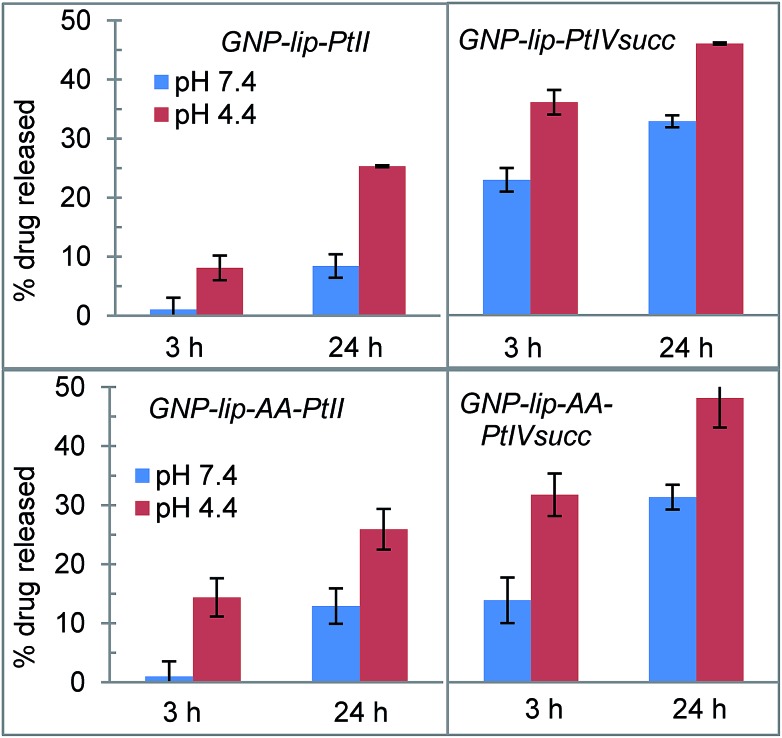
Cumulative platinum drug release from GNP-lip and GNP-lip-AA at pH 4.4 and at pH 7.4 (values and errors are presented as the mean and SD respectively from at least three independent experiments).

The percentage of drug released from each system was measured after 3 h and 24 h of incubation in buffer at room temperature. As shown in Fig. 3, in all cases drug release is significantly higher at pH 4.4 than pH 7.4. There are no major differences between GNP-lip and GNP-lip-AA in terms of release (indicating that the AA does not interfere with the Pt release) whilst in all the conditions explored, there is a higher release of PtIVsuc than PtII, suggesting a weaker binding to GNP; we speculate that a fraction of PtIVsuc molecules might not have formed coordination bonds with GNP surface carboxylate groups, and instead, could be attached solely by electrostatic interactions, with ionic strength changes effecting release.

In all cases, drug release in buffer at pH 7.4 is lower compared to release in media with similar pH (Fig. S11†), indicating that the potential Pt-binders contained in media (serum proteins, amino acids *etc.*) might play a role in facilitating the release of the drug (potentially coordinated in place of the carboxy groups that held the drug on the nanoparticle).

### Drug activity: cytotoxicity assays

The toxicity of the new nanocarrier-Pt drug conjugates was tested in the A549 human lung cancer and A2780 human ovarian cancer cell lines by MTT assay, and compared with the cytotoxicity of free drugs and nanocarriers before drug loading.

Results are summarized in Table 2, where IC_50_ values of all nanoparticle-based compounds refers to the concentrations of GNP (nM). We also calculated the concentration (μM) of Pt-drug theoretically carried by the nanocarrier at each IC_50_ point (using the drug loading studies reported in Fig. 2). This allows an approximate comparison of the activities of the conjugated and free drugs (although it does not account for any unreleased platinum that remains attached to the GNP).

**Table 2:** Cytotoxicity of the non-Pt and Pt-containing conjugates compared with free platinum drugs (72 h incubation). IC_50_ values (nM, concentration of compound needed to inhibit cell viability by 50%) of all GNP based systems refers to the concentration of nanoparticle; the corresponding concentration of Pt-drug carried by each GNP system at the corresponding IC_50_ point (*) is also reported (values and errors are presented as the mean and SD respectively from at least three independent experiments)

Compound	A2780		A549	
	IC_50_ (nM)	Pt^*a*^ (μM)	IC_50_ (nM)	Pt^*a*^ (μM)
Cisplatin	1400 ± 900		6000 ± 2200	
CisPtIVsuc-Cl	12100 ± 3700		33000 ± 12000	
GNP-lip	17.1 ± 3.7		41.6 ± 3.5	
GNP-lip-PtII	2.0 ± 0.5	4.0 ± 0.9	9.4 ± 0.6	18.8 ± 1.3
GNP-lip-PtIVsuc	4.0 ± 0.1	6.3 ± 0.1	5.5 ± 0.3	8.6 ± 0.5
GNP-lip-AA	2.6 ± 1.8		1.7 ± 0.7	
GNP-lip-AA-PtII	1.6 ± 0.6	1.9 ± 0.9	1.6 ± 0.7	2.0 ± 0.9
GNP-lip-AA-PtIVsuc	2.3 ± 0.5	1.8 ± 0.4	1.0 ± 0.7	0.8 ± 0.7

^*a*^Concentration of Pt-drug (μM) potentially carried by the GNP at IC_50_ concentration.

For the GNP-lip carrier, in the case of the Pt(ii) conjugates the IC_50_ values are similar to those of cisplatin alone (see ESI Table 1† for statistical analysis). However delivery of Pt(iv) drug using GNP-lip system does enhance the activity of the drug as concentration of the drug carried at IC_50_ points is 2 and 4 fold lower (in A2780 and A549 respectively) compared to IC_50_ values of the free drug; this is likely due to a combination of the drug amplification effect (typical for drug delivered by nanocarriers) and the higher drug release of this system (compared to the Pt(ii) release, see Fig. 3 and S11†).

Our experiments in these cell lines do reveal a certain level of toxicity for GNP-lip systems even though such nanoparticles have recently been described as non-toxic in different biological systems and at lower incubation times.^49^ Nevertheless, the toxicity of each of the drug conjugates is higher than that of the carriers alone. The design offers potential to but does not yet include additional targeting vectors for specific cells or tissues,^4^,^25^–^29^ but would potentially benefit from gold nanoparticle enhanced permeability and retention *in vivo*.

As envisaged in our design, the GNP-lip-AA nanocarrier showed a higher toxicity compared to the non AA-containing system, GNP-lip; anti-proliferative activities of several aminoanthraquinone derivatives has been described and they can work as DNA intercalators or groove/backbone binders and/or are able to generate reactive oxygen species upon visible light illumination (although it should be noted that cells were incubated in the dark).^50^,^51^ Indeed, the presence of AA on these nanocarriers does affect binding to DNA (DNA binding studies were performed and discussed below). The GNP-lip-AA species seem to have particularly good activity. The interpretation of the data for the conjugates is complicated by the plethora of species that will potentially be present and active (partially unloaded GNP-lip-AA-Pt species as well as released platinum drug; possibly also some fully unloaded GNP-lip-AA) but it is clear that both the Pt(ii) and Pt(iv) conjugates have high activity while no statistically significant advantage over the activity of the GNP-lip-AA carrier itself is detected. Hambley has reported a mononuclear cisplatin linked to an anthraquinone and similarly noted being unable to statistically distinguish the cytotoxicity from that of the anthraquinone.^52^,^53^ We will return to consider this activity after first exploring the uptake and localisation of these nanoparticles inside the cells.

### Nanoparticle uptake in cells: confocal microscopy

Confocal laser scanning microscopy (fluorescence, reflection and transmission microscopy) was employed to visualize the nano-conjugates inside cells. Non-fluorescent GNP-lip particles could be imaged only by transmission (brightfield mode) and reflection, whilst fluorescent signals of AA-containing particles could be detected using excitation at 488 nm. Since particles are about 15 nm in size, it should be noted that only particles forming aggregates inside the cells will be resolved and therefore visualized by the reflectance technique.^54^

**Fig. 4:**
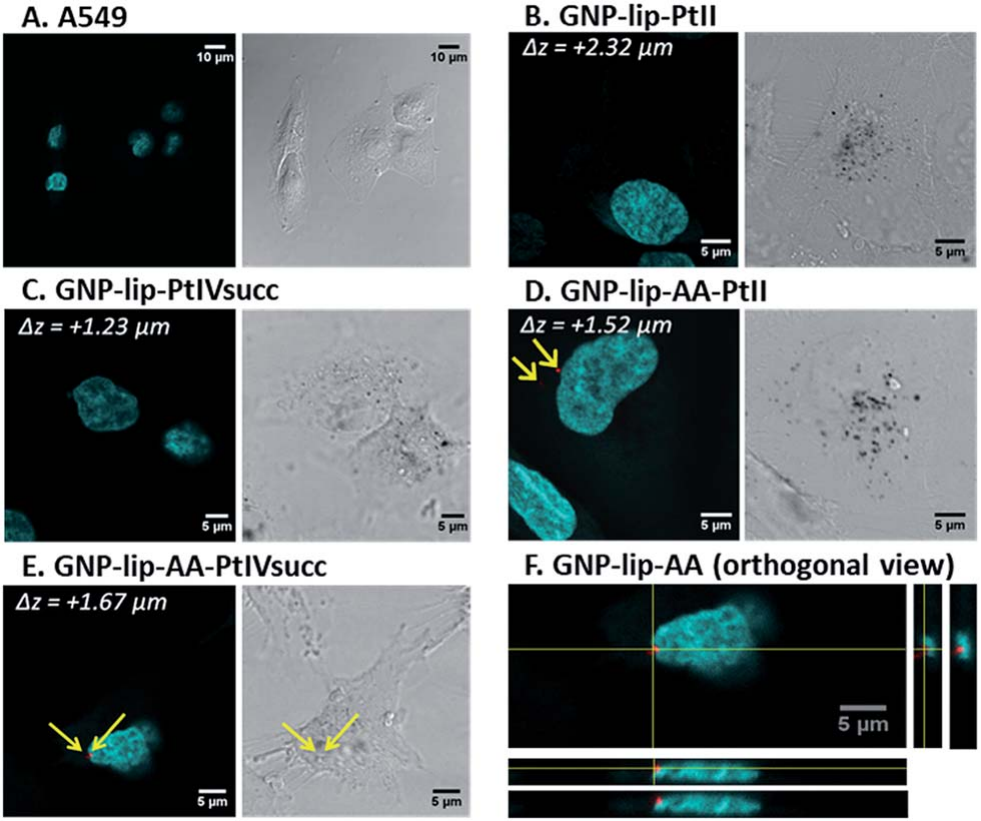
Confocal microscopy images of A549 alone (control A) and treated with different nanocarrier conjugates (B to E) for 24 h, followed by nuclei staining with Hoechst 33258 and fixation. Each image (A to E) shows (i) on the left, merged fluorescent images of nuclei stained by Hoechst 33258 (cyan) and AA (red, present only in (D and E), indicated by yellow arrows), obtained upon excitation at 405 and 488 nm respectively; (ii) on the right, the corresponding transmission images, where GNPs appear as black dots. In (F), one example of orthogonal view, upon compiled from a *Z*-stack of fluorescent images (Hoechst overlayed with AA channel) to confirm that AA fluorescence is inside the cell at the perinuclear region.

A459 and A2780 cells were treated with each compound for 3 and 24 hours, followed by nuclear staining with Hoechst 33258 (cyan) and fixation. Representative examples of fluorescent and transmission images are reported in Fig. 4 and further images are presented in Fig. S12–S37† showing early uptake (from 3 h). Overall, we observed cellular uptake of all nanocarriers, particularly evident as black dots in transmission images. The uptake at the 24 hour time point is significantly higher although particles were present in cells already after 3 hours. No differences were observed between the two cell types nor when varying GNP types (either loaded or not with drugs), suggesting that the loaded drug does not affect the cellular uptake of the system. No changes in cell morphology or disruption to the nucleus were evident (Fig. 4).

All GNPs were located mainly in the cytoplasm and in the perinuclear region, rather than penetrating into the nucleus, suggesting that their cytotoxic activity is either due to drugs being released and/or a different type of action occurring into the cytoplasm. It must be noted however, that single/non- clustered nanoparticles would not be visible, therefore their presence in other parts of the cell (*i.e.* nucleus) is not excluded. *Z*-stack images were recorded and orthogonal views confirm that particles were indeed located inside (and not just on the surface of) cells (Fig. 4F and S14–S37†). For all AA-containing GNPs, signals corresponding to AA were evident in all images, often in the perinuclear area, however signal intensity was rather modest (*i.e*. red dots in Fig. 4D, E and F) and may be attributed only to where nanoparticles have accumulated/clustered sufficiently.

### Nanoparticle uptake: transmission electron microscopy

**Fig. 5:**
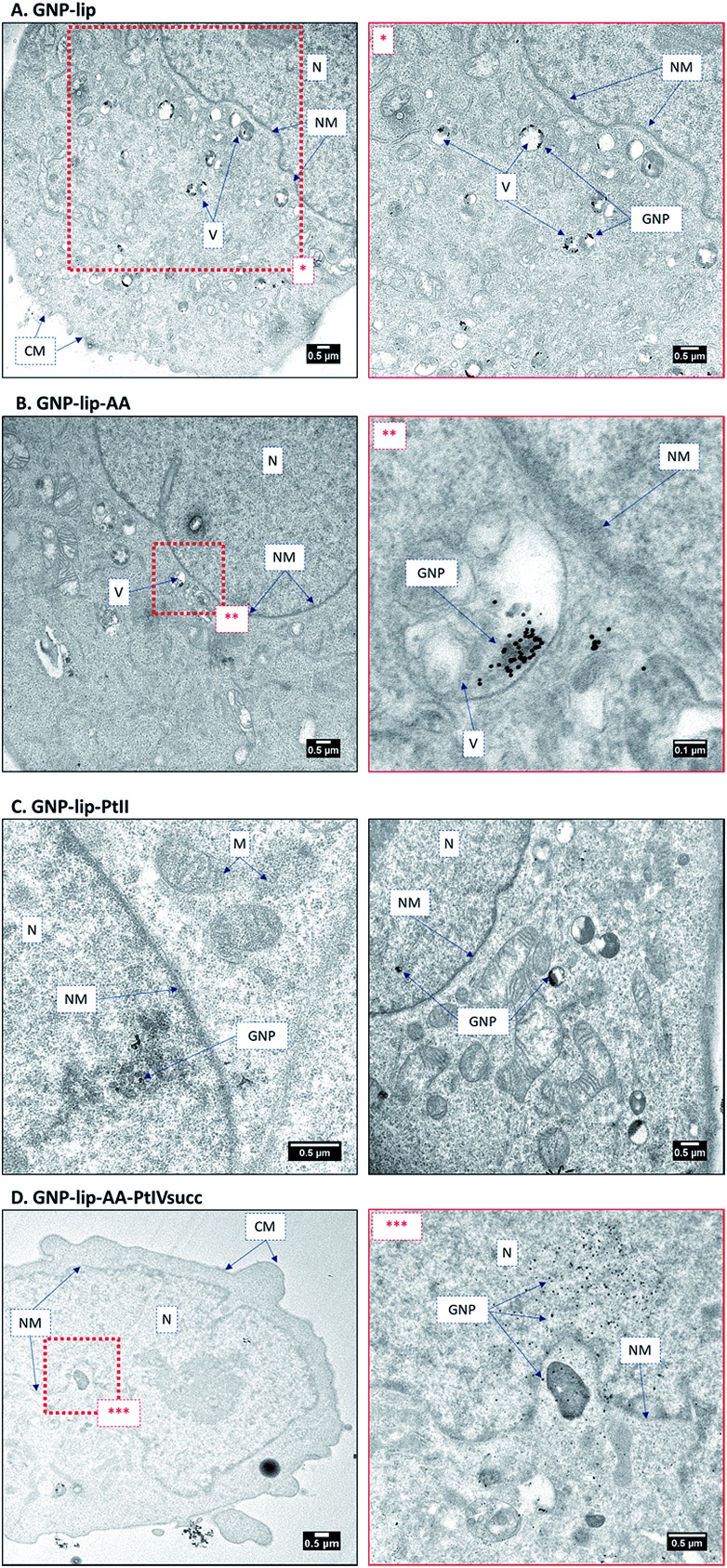
TEM images of A549 treated with (A) GNP-lip, (B) GNP-lip-AA, (C) GNP-lip-PtII, and (D) GNP-lip-AA-PtIVsuc for 24 hours (N: nucleus, NM: nuclear membrane, CM: cell membrane, V: vesicles, M: mitochondria). Magnifications of red-dashed inset boxes are presented in the images labelled with corresponding asterisks.

To ascertain the intracellular localization of the nanoconjugates, A549 and A2780 cells were treated for 24 h with the nanoparticles and then imaged by TEM. Inside the cells most particles were found to accumulate in endosomal vesicles in the perinuclear region (representative examples in Fig. 5 and further images in S38–S44). One possible mechanism of delivery is *via* a vesicular intermediate directly from the plasma membrane. Some micrographs also show a few particles freely dispersed in the cytosol, in the endoplasmic reticulum and inside the nucleus. This implies that these nanocarriers can circumvent, or escape from, the endosomal pathway and end up in the nucleus without the need for specific functionalization, such as addition of membrane penetrating peptides.^54^

In such cases a fraction of platinum drug would potentially be delivered into the nucleus attached to the GNP, while the remainder would be released in the cytosol and make its way freely towards the nucleus. In all cases, the particles show signs of clustering when they are inside to the endosomes, consistent with the confocal images where only aggregates could be detected. In general, a higher vacuolization was observed, compared to non-treated cells, and even nuclear disintegration when A2780 cells were treated with GNP-lip-AA (Fig. S42†).

### Interaction of GNP-lip-based nanocarriers with ct-DNA

Given the higher cytotoxic activity of GNP-lip-AA (compared to the AA-free nanocarrier GNP-lip) and the observation of the nanoparticle penetration into the nucleus, to better understand the nanocarrier effect, we compared the binding properties of the two unconjugated nanocarriers to calf thymus (ct) DNA by flow linear dichroism (LD), circular dichroism (CD) and Dynamic Light Scattering (DLS) studies.

**Fig. 6:**
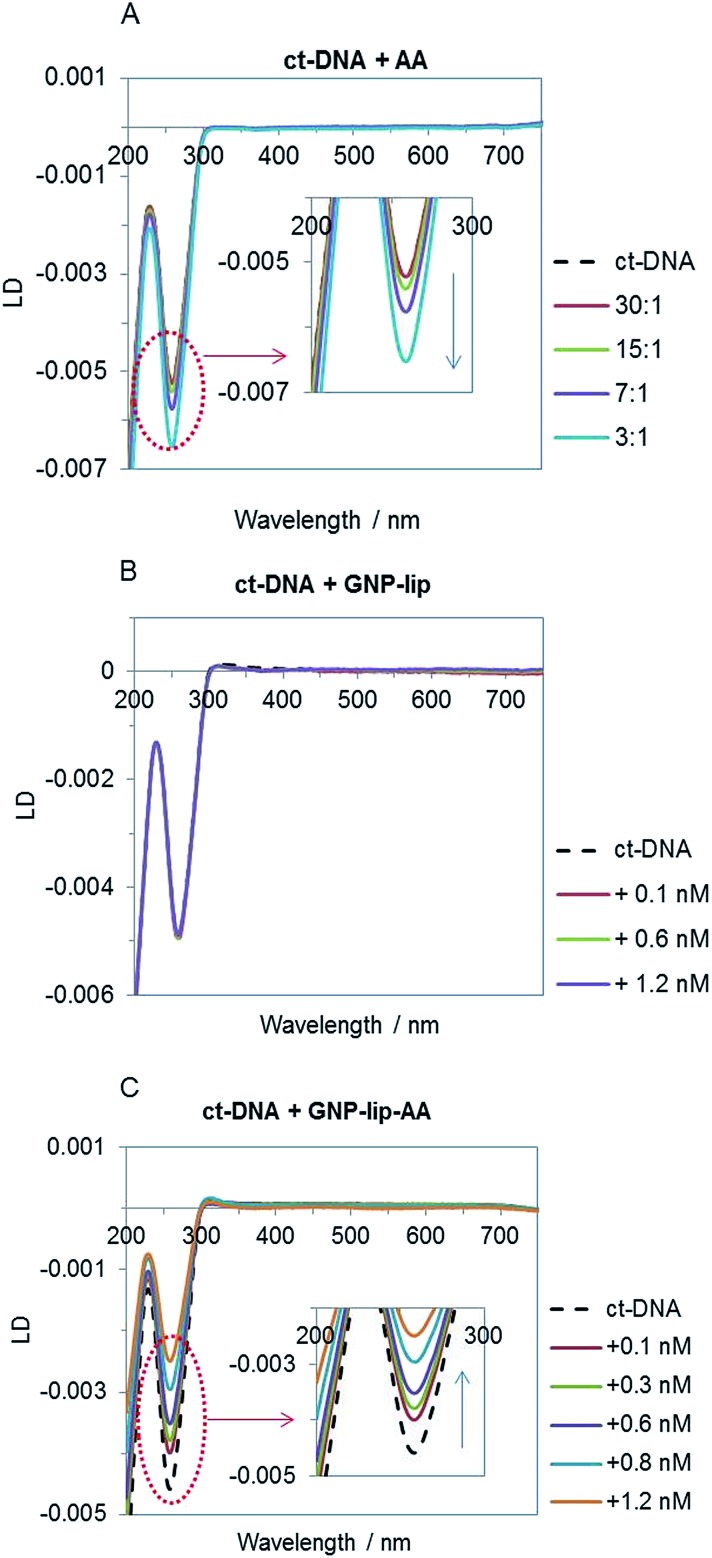
Linear dichroism spectra of ct-DNA (75 μM in Tris–HCl 1 mM and NaCl 10 mM, pH 7.5) titrated with AA (A) GNP-lip (B) and GNP-lip-AA (C). Legends show ct-DNA : AA ratio in (A) and final concentration of nanocarriers in (B) and (C).

All experiments were performed in NaCl (10 mM) and Tris–HCl (1 mM, pH 7.5) buffer; no particle aggregation and/or ct-DNA precipitation was observed in these conditions (controls by UV-vis in Fig. S45†). The LD signal of ct-DNA (negative band at 260 nm) decreases upon addition of increasing concentration of the free aminoanthraquinone derivative AA (Fig. 6A) as would be expected; this is typically observed when DNA stiffening is caused by the presence of intercalative agents. GNP-lip alone does not affect DNA orientation in flow (Fig. 6B) whilst GNP-lip-AA causes an increase of LD signal. Such an increase is typically observed with coiling and/or bending of DNA which causes a reduction in the linear orientation of the DNA in the flow (and thus a signal reduction).^72^ These results indicate that presence of AA is necessary to achieve binding between the nanocarrier and DNA. Since the free AA binds by intercalation and that intercalating part of the AA can project away from the nanoparticle surface and towards solution, it seems likely that the presence of multiple intercalating units anchored on single nanoparticles is resulting in a ‘DNA wrapping’ effect around the nanoparticle. The length of ct-DNA implies this condensation might involve more than one nanoparticle per DNA. The binding and coiling effects are remarkable since both DNA and the GNP-lip-AA are negatively charged. There is precedent for condensation of DNA by cationic polymers and nanoparticles (and indeed this is used in gene delivery systems) but this is through electrostatic attraction, with backbone charge compensation facilitating the DNA bending.

Titrations of ct-DNA *vs.* GNPs were performed also by circular dichroism (CD); the spectra collected confirmed that the GNPs cause no significant perturbation of B-DNA structure (*i.e*. a basic DNA structure is retained in the DNA coiled around the GNP-lip-AA). No induced CD signals in the GNP spectroscopies were observed (data not shown).

**Fig. 7:**
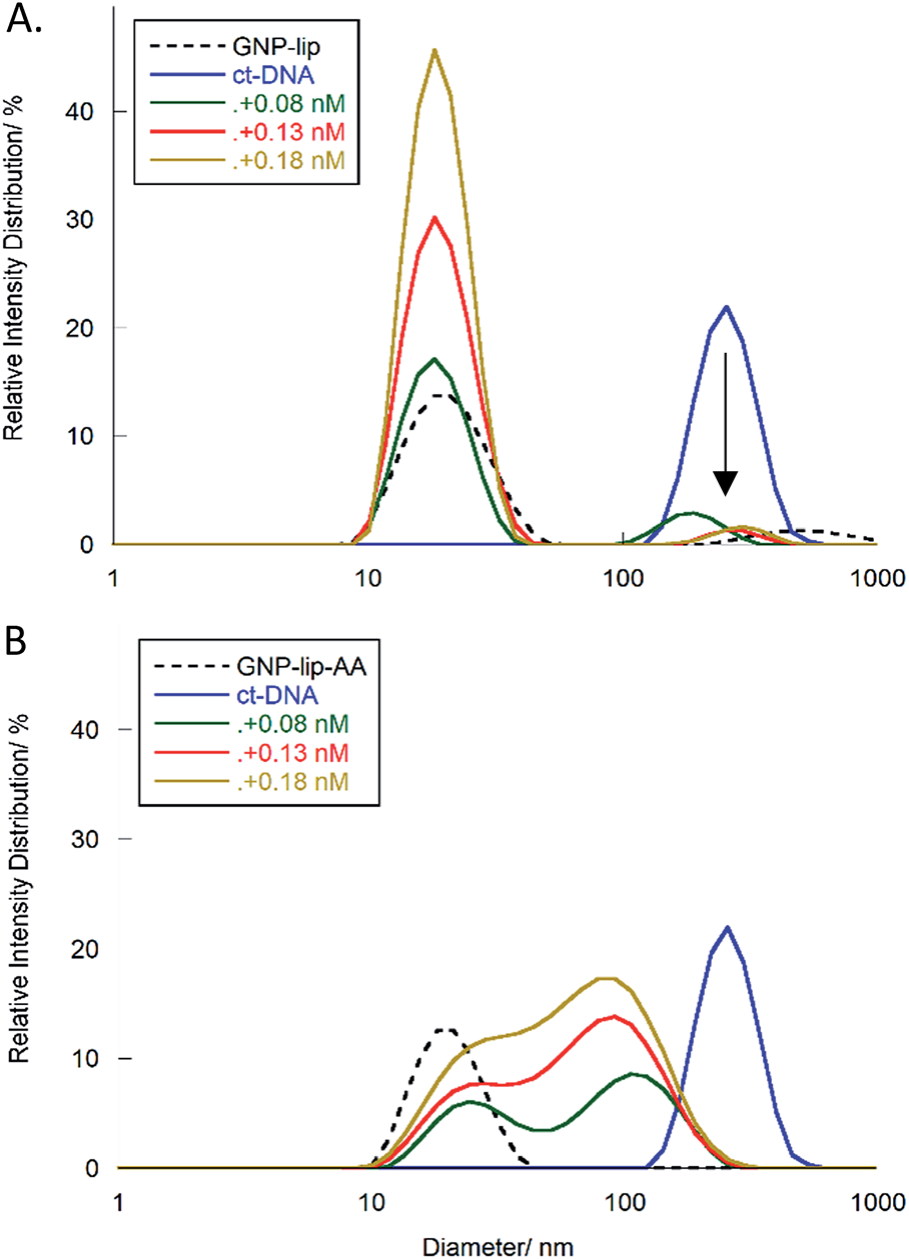
DLS histograms showing the relative size distribution of ct-DNA (11 μM, in Tris–HCl 1 mM and NaCl 10 mM), with an increasing concentration of (A) GNP-lip and (B) GNP-lip-AA. Controls corresponding to ct-DNA alone and GNPs alone are in both graphs as blue solid and black dashed lines respectively.

To provide confirmation of the interpretation of the LD results, we further studied the interactions between ct-DNA and the GNPs by DLS. Histograms in Fig. 7 report the percentage of species present in one sample (expressed in relative intensity distribution) with specific sizes (diameter in nm). ct-DNA alone (blue lines) shows a unimodal peak centered at 250 nm, whilst peaks corresponding to GNP-lip or GNP-lip-AA alone (black dashed lines in Fig. 7A and B respectively) are centered around 19 nm.

Samples containing ct-DNA and GNP-lip (Fig. 7A) produce two signals corresponding to separate GNP (18 nm; unchanged by concentration) and DNA species (∼250 nm). The DNA species are slightly shifted between concentrations but there is no evidence of new species with different sizes suggesting that no significant changes in DNA conformation and/or GNP-lip aggregation, and/or binding between the species, occurs.

Conversely, addition of GNP-lip-AA to the DNA (Fig. 7B) causes the loss of the DNA signal around 250 nm and appearance of a distinct bi-modal size distribution signal, which includes (i) a peak at 24 nm, that remains at different concentrations of GNP and (ii) a larger peak that shifts from 108 nm to 93 nm to 84 nm with increasing concentration of GNP. The peak at 24 nm could either correspond to a new GNP-DNA adduct or to GNP-lip-AA alone, size-shifted because of the different ionic strength of the system. The size of the larger species decreases when more GNP-lip-AA is added and would therefore correspond to increased coiling/wrapping of the DNA as more GNP-lip-AA is added and binds: this is consistent with, and reinforces, the observations from LD studies (Fig. 6C). Of the two nanoparticles types, only GNP-lip-AA displays binding and coiling activity toward DNA and it seems reasonable to hypothesise that this may be related to its higher cytotoxicity.

**Fig. 8:**
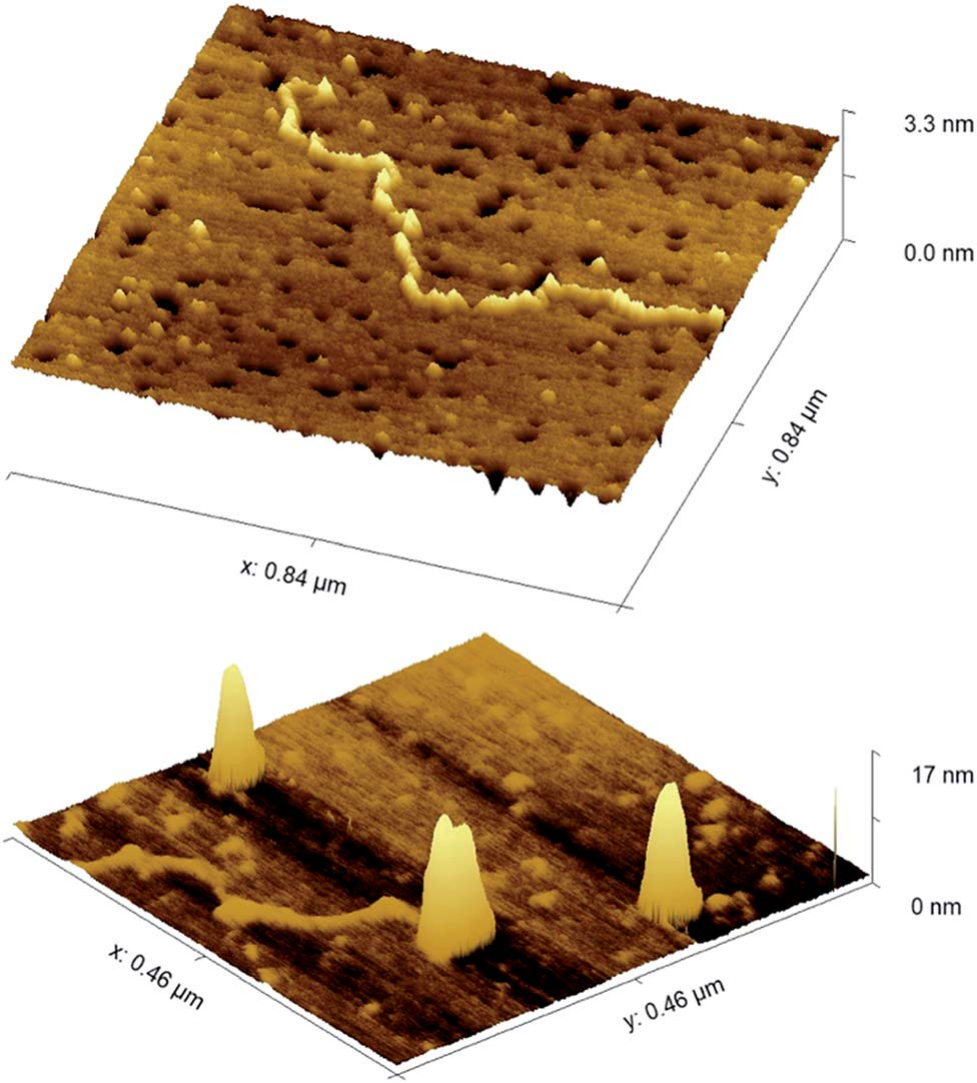
AFM images of linearised pBR322 plasmid -DNA, (top) alone and (bottom) with 2 equivalents of GNP-lip-AA. 2D images are included in Fig. S46.†

We have also explored the coiling/wrapping using AFM imaging. In contrast to the LD and DLS solution techniques, AFM requires surface immobilisation; it was challenging to find a suitable surface coating that gave artefact free results. APTES (an amine-based surface popular for DNA and nanoparticle imaging) allowed deposition but in our hands, showed some tendency to promote DNA condensation in absence of nanoparticles. However using nickel coated mica, we were able to image linearised plasmid pBR322 (4361bp) alone and with two equivalents of GNP-lip-AA. As shown in Fig. 8, the DNA does indeed wrap about the nanoparticles. In the centre of the image a partially wrapped-up DNA is observed, with two nanoparticles observed wrapped within this DNA at one end and the other end of the DNA flowing out from the particles. The width and shape of the nanoparticles clearly confirms that DNA is wrapped about them. The broadening at the base of the structures is likely a consequence of the surface deposition rather than an indication of some asymmetry in the wrapping in solution. While the solution techniques alone are compelling evidence of the wrapping of the DNA around these anionic nanoparticles, the AFM provides further evidence for this remarkable binding and DNA wrapping.

Cationic nanoparticles are known DNA condensation agents^55^ driven by strong electrostatic attraction to the DNA polyanion, however anionic nanoparticles will not benefit from such strong electrostatic interaction energy (unless under high salt conditions where salt cations might shield the anionic repulsion). Nevertheless, in intriguing early studies, Murphy^56^ and Åkerman^57^ reported some form of interaction between ds-DNA and citrate coated gold nanoparticles and there have been some subsequent similar reports.^33^,^58^,^73^–^75^ The interaction is weak and its nature unclear, with suggestions including direct adsorption to the gold with displacement of the citrate.^56^ This does demonstrate that anionic charge on the surface of the nanoparticle does not exclude DNA-binding as long as there are sufficient favourable interactions to counter it; on a macromolecule such as a nanoparticle this may be possible. However, such ds-DNA binding is not replicated by our anionic lipoic GNP-lip particles (without the AAs) under the conditions of our studies; no indication of a significant DNA interaction is observed (by CD, LD or DLS). The binding of the AA functionalised nanoparticles (GNP-lip-AA) to DNA is thus not simply an inherent property of lipoic coated gold nanoparticles, but a consequence of the AA groups on the surface.

The most common examples of intercalators on gold nanoparticles are the many reports of doxorubicin functionalised gold nanoparticles, inspired by the doxorubicin liposomal formulations that are enjoying success in the clinic.^59^–^64^ Yet these designs are all focused on using cleavable linkers to attach to the nanoparticle surface so as to facilitate release of the doxorubicin; the DNA-binding of these particles themselves has not been explored. In just a few reports intercalators have been attached to gold nanoparticles for example to detect DNA in assays: Murray attached low loadings of ethidium bromide (1–2 per nanoparticle) to cationic and anionic gold nanoparticles through a C12 chain thiol.^65^ The cationic nanoparticles bound rapidly, but the anionic ones only slowly and in high salt suggesting the binding was salt assisted. Subsequent work has attached acridine orange through alkylthiol chains onto neutral PEG coated gold nanoparticles or *via* attachment to a chitosan polymer, and psoralen *via* a high-molecular-weight aminodextran polymer.^66^–^68^ All bind DNA, but the designs are each distinct from that herein, and none of these reports explored or noted DNA coiling effects.

Our findings have implications for the interpretation of the activities of designs used for GNP (and indeed other nanoparticle) delivery of doxorubicin. Many of these nanoparticle delivery systems report enhanced doxorubicin activity. The assumption has been that the increased activity is due to better target delivery of active doxorubicin released from its nanocarrier, and release of large payloads in the same vicinity. Yet when using cleavable linkers, a range of species is possible in solution, including not only the free doxorubicin, but nanoparticles with varying numbers of residual doxorubicins. The work herein, indicates that such nanoparticles can bind DNA in new ways different from the individual drug, and can cause distinct and potent effects. If nanoparticles bearing residual (un-cleaved) doxorubicins penetrate the nucleus or mitochondria to encounter cellular DNA, or encounter RNA elsewhere in the cell, they could be acting as non-innocent species adding enhanced toxicity beyond that of the released drug itself. This brings an intriguing new dimension to how we could consider such agents.

## Experimental details

All gold nanoparticle suspensions were stored in darkness at 4 °C and filtered (glass fibre syringe filter 1 μm) before being used/characterized. Deionised water was used in all syntheses. 14 nm citrate gold nanoparticles were prepared according to previously reported protocols.^69^

^1^H NMR and ^13^C NMR spectra were recorded on ACIII-300 (300 MHz) and AVIII-400 Bruker spectrometers (400 MHz), respectively. Chemical shifts were referenced to tetramethylsilane or H_2_PtCl_6_ as an internal standard or to residual solvent peaks. Electrospray ionization (ESI) mass spectra were recorded on a Micromass LCT ToC mass spectrometer in a positive ionisation mode. FT-IR spectra were recorded on a Nicolet Avatar 320 spectrophotometer with an ATR attachment. UV-vis analyses were carried out at room temperature in a Varian Cary 5000 UV-vis spectrophotometer using 10 mm cuvettes. The hydrodynamic diameter and surface charge (*ζ*-potential) of the nanoparticles were measured using a Zetasizer Nano ZS (Malvern Instruments Ltd, Malvern, UK). Measurements were performed (12 cycles per run) in triplicate. The value for the hydrodynamic diameter of the particles was obtained using the number scaling.

Fresh Pt-functionalized samples were used for each measurement and cell experiment. Each spectroscopic (UV-vis, LD, DLS) experiment was repeated at least twice to confirm reproducibility.

All reported uncertainties represent one standard deviation calculated from at least three replicate measurements.

### Synthesis of platinum drugs

Cisplatin was purchased from Alfa Aesar and used without further purification. The Pt(iv) complex *c*,*c*,*t*-[Pt(NH_3_)_2_Cl_2_(OH)(O_2_CCH_2_CH_2_CO_2_H)] (PtIVsuc-Cl) was prepared as previously described.^44^ Afterwards chloride ions from both complexes were removed by adding AgNO_3_ to form AgCl precipitate. The solutions were filtered (0.45 μm filter) and then centrifuged to remove residues of AgCl. Finally they were diluted to obtain the working solutions of [Pt(NH_3_)_2_(H_2_O)_2_](NO_3_)_2_ (PtII–H_2_O) and [Pt(NH_3_)_2_(H_2_O)_2_(OH)(O_2_CCH_2_CH_2_CO_2_H)](NO_3_)_2_ (PtIVsuc-H_2_O). Accurate determination of the concentration of working solutions was carried out by ICP-MS analysis.

### Synthesis of 1-amino-5-(1,8-dimercapto-3,6-dioxaoctanyl)-anthracene-9,10-dione (AA)

A similar procedure to that described for the non-amino anthraquinone analog was followed.^70^ 1-Amino-5-chloroanthraquinone (1.43 g, 5 mmol) and 2,2′-(ethylenedioxy)diethanethiol (1.92 g, 10 mmol) were mixed with NaH (60% oil dispersion, 0.80 g, 20 mmol) in anhydrous THF (100 mL), and the mixture was stirred for 3 h at room temperature under argon. The mixture was poured into ice-water (200 mL), and acidified carefully to pH 5 by adding HCl 1 M. The solution was extracted with CH_2_Cl_2_ (4 × 50 mL), the organic solution was washed with water (2 × 50 mL) and dried over sodium sulphate. The solvent was evaporated at reduced pressure, and the dark-red residue was purified by column chromatography (silica gel, CH_2_Cl_2_: ethyl acetate, 98 : 2, v/v). Rf 0.3. Yield 50%. ^1^H NMR *δ*, ppm (CDCl_3_): 3.88 (t, e5, 2H, *J* = 6.9 Hz), 3.68 (m, e2/e3/e4, 6H), 3.27 (t, e6, 2H, *J* = 6.96 Hz), 2.73 (dd, e1, 2H, *J*1 = 8.22 Hz, *J*2 = 6.45 Hz), 1.62 (t, 1H, SH, *J* = 8.19), 6.84 (NH_2_), 6.96 (dd, a2, 1H, *J* = 8.34 Hz, *J* = 1.11 Hz), 7.50 (t, a3, 1H, *J* = 7.88 Hz), 7.67–7.70 (m, a4/a7/a8, 3H), 8.15 (dd, a6, 1H, *J*1 = 6.00 Hz, *J*2 = 2.88 Hz). ^13^C NMR *δ*, ppm (CDCl_3_): 135.0 (a3), 132.9 (a7), 128.8 (a8), 123.1 (a6), 122.3 (a2), 117.5 (a4), 73.0 (e2), 70.6, 70.2 (e3, e4), 69.1 (e5), 31.6 (e6), 24.3 (e1). See ^1^H NMR and ^13^C NMR spectra in Fig. S1.†

ESI-MS(+), *m*/*z*: 426.3 (M + Na)^+^. IR (cm^–1^): 3460 (O–H), 3344 (S–H), 2890 m (N–H). The compound AA is soluble in acetone, ethanol, methanol, CHCl_3_, CH_2_Cl_2_, THF and DMSO, but it is not soluble in water. *ε* (480 nm, ethanol) ∼9100 M^–1^ cm^–1^. Luminescence in ethanol: *λ*_exc_ 480 nm, *λ*_em_ 590 nm.

### Preparation of GNP-lip

A modified protocol from that previously described by Lin *et al.* was followed.^71^ A volume of 80 μL of NaOH 1 M was added to 16 mL of 14 nm-citrate GNPs and 1–2 min later, 1.6 mL of an ethanolic solution of (±)-α-lipoic acid (10 mM) was added. The mixture was stirred at room temperature for 4 h in darkness. The solution was further purified by centrifugation at 13000 rpm for 30 min. Supernatants were discarded and the pellets were re-suspended in Milli-Q® water. Finally 108 μL of NaOH 1 M were added to stabilize the suspension. Final concentration (ICP-MS): 6 nM. IR (cm^–1^): 1690 m (C=O), 1456 s.

### Preparation of GNP-lip-AA

Direct functionalization of citrate GNPs with AA ligand was attempted but, the resulting particles aggregated spontaneously, probably due to the absence of electric surface charge. Therefore, stable negatively-charged GNP-lip particles were partially substituted with AA to yield stable and negatively-charged nanoparticles with a mixed surface.

25 mL of 400 μM AA ethanolic solution was added to 100 mL of a GNP-lip solution (3 nM) and stirred overnight (18 h) in darkness and at room temperature, followed by washing with chloroform until the washing solution was colorless. The remaining chloroform was removed by rotary evaporation.

The solution was dialyzed (Spectra-Por® Float-A-Lyzer® G2, 5 mL, MWCO 8–10 kDa) for 2 days in deionized water with NaOH 1 M (0.5 mL/2 L) pH 10–11 and water changed every 6–8 hours.

The solution was filtered and stored at 4 °C in the dark. The absence of unbound AA is proved by the narrowing of the SPR band and its shift to 528 nm (disappearance of the convoluted band at 480 nm). Also, the band intensity around 240 nm decreases significantly. To ensure the absence of non-covalently bound AA, periodic washings with chloroform were performed and checked by UV-vis. Final concentration (ICP-MS): 2.5 nM. To stabilize the colloidal suspension, NaOH 1 M was added until pH ≈ 11. IR (cm^–1^): 1685 s (C=O, anthraq), 1639 s (C=O, lip), 1420 m, 1055–1274 m, (aromatic C=C, C=N, anthraq) (Fig. S8†). Luminescence in water: *λ*_exc_ 470 nm, *λ*_em_ 645 nm. GNP-lip and GNP-lip-AA are both stable against precipitation in water over a period of at least 3 months at 4 °C.

### Attachment of platinum complexes to gold nanoparticles

To attach either PtII–H_2_O or PtIVsuc-H_2_O, the corresponding aqueous platinum solution (4.8 mM for PtII–H_2_O, 3.7 mM for PtIVsuc-H_2_O) was added slowly to the particle solution (3 nM for GNP-lip, 2.5 nM for GNP-lip-AA) in the following molar ratios GNP:Pt: 1 : 13 × 10^3^ (GNP-lip-PtII), 1 : 10 × 10^3^ (GNP-lip-PtIVsuc), 1 : 6 × 10^3^ (GNP-lip-AA-PtII), 1 : 4.5 × 10^3^ (GNP-lip-AA-PtIVsuc). In each case, the mixture was brought to pH 10.3 with NaOH 1 M and stirred overnight in darkness for 18 h. Then, the solution was filtered and purified by centrifugation at 5000*g* for 30 min and further re-dispersed in NaOH 25 μM (pH 8.5). The selection of 5000*g* was made experimentally as higher speeds led to problems with redispersion. Adding further centrifugation steps, or brief (<2 h) dialyses in 25 μM NaOH, didn't affect loading as assessed by ICPMS.

### Drug release studies

Release studies in physiological media. Freshly-prepared and purified Pt-functionalized nanoparticle suspensions (NaOH 25 μM, pH 8.5) were centrifuged at 5000*g* for 30 min and the pellets re-dispersed in supplemented RPMI-1640 or DMEM cell media (purchased by Gibco). The suspensions were incubated at 37 °C in darkness. Sample aliquots were taken over time and centrifuged to remove the supernatants containing the released drug. Pellets were re-dissolved in clean cell media and digested (see below) for ICP-MS analysis.

Both platinum and gold concentrations were determined simultaneously. Cumulative release percentage was obtained according to the following formula:
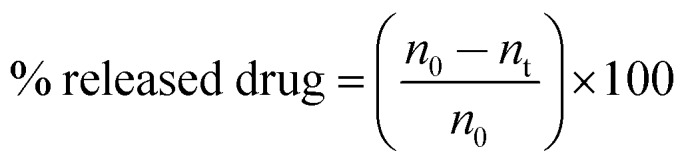
where *n*_0_ is the initial number of platinum atoms (from the drug) per particle and *n*_t_ is the number of platinum atoms at a certain time interval. Two independent measurements were carried out.

### pH-dependent release studies

Freshly prepared and purified Pt-functionalized nanoparticle suspensions (NaOH 25 μM, pH 8.5) were centrifuged at 5000*g* for 30 min and the pellets re-dispersed in phosphate buffer saline (pH 7.4), or in phthalate buffer (pH 4.4). The solutions were kept at room temperature and darkness. Aliquots were collected after 3 h and 24 h, immediately centrifuged and resulting pellets were re-dispersed in clean buffers. Gold and platinum concentrations were determined simultaneously by ICP-MS. The percentage of released drug was obtained in the same way as that used to obtain the cumulative release in cell media (*vide supra*).

Two independent measurements were carried out.

Phthalate buffer pH 4.4 was prepared by adding 20 mL of 0.1 M NaOH to 100 mL 0.1 M potassium hydrogen phthalate.

### ICP-MS analysis

Samples were digested with aqua regia at 80 °C overnight, then diluted to 2% aqua regia solutions and filtered. Platinum and gold standards (Fluka) were prepared containing 2% aqua regia.

Gold and platinum (^195^Pt) concentrations were simultaneously determined in an Agilent 7500CX ICP-MS (Pt simple cone in no-gas mode). Analysis conditions: Ar flow: 15 L min^–1^, auxiliar gas 0.9 L min^–1^, make-up gas 0.15 L min^–1^, RF power 1550 W, RF matching 1.8 V, analog HV 1750 V, Pulse HV 1130 V, spray chamber temperature 15 °C, nebuliser pump 0.08 rps. Internal standard (50 ppb Er solution) is introduced into the sample flow through a T-piece. All readings were in triplicate. Results are means ± s.d. of three independent experiments.

Concentration of gold measured by ICP-MS combined with the known size of particles from TEM characterization allows calculation of particle concentration.

### Cell culture

Human ovarian carcinoma A2780 and human lung carcinoma A549 cell lines were obtained from The European Collection of Authenticated Cell Cultures (ECACC) operated by Public Health England (A549 cat number 86012804; A2780 cat number 93112519) and maintained in Roswell Park Memorial Institute medium (RPMI-1640) (GE Healthcare) and Dulbecco's Modified Eagle Medium (DMEM) (Gibco), respectively, in both cases supplemented with FBS (10%, v/v), penicillin (50 U mL^–1^ culture medium), streptomycin (50 μg mL^–1^ culture medium) and l-glutamine (2 mM). Cells were grown in a humidified incubator at 37 °C, 5% CO_2_, and 95% relative humidity. Cells were routinely sub cultured using a 1 in 4 dilution using a standard trypsin–EDTA protocol.

### MTT cell proliferation assay

Human A2780 and A549 cells at logarithmic growth phase (6 × 10^4^ and 5 × 10^4^ cells per mL, respectively) were added to 96-well culture plates, respectively, at 100 μL per well, and incubated overnight to allow attachment.

Medium was removed and replaced with 100 μL of medium containing sample at different concentrations (four replicates per sample), and the plates were incubated at 37 °C in 5% CO_2_, 95% relative humidity for 72 hours. Cells were thoroughly washed with PBS before adding 100 μL of thiazolyl blue tetrazolium bromide (0.5 mg mL^–1^). The cells were further incubated for 2 h. The media was carefully removed by aspiration and 200 μL of DMSO added to dissolve the purple crystals. Absorbance was measured using a 96-well plate reader set at 590 nm. Non-treated cells were used as a control. Results are the mean ± SD of at least three independent experiments. Concentration of GNP and platinum of assayed sample solutions were accurately determined by ICP-MS. Light microscopies and TEM images indicated cell death and not just cytostasis.

### Confocal Microscopy

A2780 and A549 cells were seeded in 6-well plates onto sterilised circular glass slides of 2 cm diameter at 40% confluence and left incubating overnight to attach. Next day, cells were washed with PBS, treated with 0.6 nM solutions of nanoparticles and further incubated at 37 °C for 3 h and 24 h. For the last 30 min of each incubation period, cells were treated with Hoechst 33258 at a final concentration of 4 μM for nucleus-staining and left incubating at 37 °C. Then cells were washed with PBS and fixed with 4% paraformaldehyde for 15 min, followed by washing with PBS (2×) to remove paraformaldehyde. Coverslips were mounted onto microscope slides using non-fluorescent aqueous Hydromount (National Diagnostics, UK) as mounting solution. Samples were imaged on a Leica SP2-AOBS Inverted Confocal Laser Scanning Microscope with an oil immersion objective HCX PL APO lbd.BL 63.0 × 1.40 OI. Fluorescence images were taken using the laser lines 405 nm (Hoechst) and 458-476-488–496 nm (GNP-lip-AA). For transmission and reflection images, 405 nm and 488 nm laser lines were used respectively. Images were processed by ImageJ software (http://rsbweb.nih.gov/ij/).

### Transmission electron microscopy

For characterization of nanoparticles, a JEOL2100 TEM, 200 keV and Gatan multiscan camera was used. Cell uptake samples (24 h) were imaged using JEOL1200EX TEM, 80 keV and Gatan multiscan camera. Cells were seeded in 6-well plates onto sterilised circular glass coverslips of 1 cm diameter at 30% confluency and left incubating overnight to attach. Next day, cells were washed with PBS, treated with 0.6 nM solutions of nanoparticles and further incubated for 24 h. Afterwards the slides were washed with PBS and submerged in glutaraldehyde/formaldehyde (2.5%, 0.5 M PBS). Cells were subsequently placed in 1% osmium tetroxide solution and gradually dehydrated with alcohol and propylene oxide. Then they were embedded in resin and ultrathin sections (50–150 nm) obtained which were mounted onto copper electron microscope grids (Formvar film). Samples were contrasted with uranyl acetate and lead citrate.

TEM images were acquired using Digital Micrograph 1.8 (Gatan, CA, USA) and post-imaging analysis was performed using ImageJ.

### DNA-binding studies

Both CD and LD titration were performed by adding increasing concentration of compound (GNP and AA from stock solutions in water and ethanol respectively) to calf thymus (ct) DNA (75 μM in NaCl 10 mM and Tris HCl 1 mM pH 7.5). Equal volume of ct-DNA 150 μM was added at each addition of compound, to keep DNA concentration constant. ct-DNA (supplied by Sigma-Aldrich) concentration was determined by UV-vis using the molar extinction coefficient *ε*_258_ = 6600 M^–1^ cm^–1^.

CD spectra were recorded at 20 °C on a Jasco J-810 spectropolarimeter whilst LD experiments were performed using a Chirascan-plus instrument by Applied Photophysics provided of the High Shear Couette Cell Accessory (CCA).

All dynamic light scattering (DLS) measurements were performed using a Malvern Zetasizer Nano ZSP, using a 4 mW He–Ne laser with an operating wavelength of 633 nm. For size measurements, temperature was maintained at 25 °C with an equilibration times of 120 before a measurement was performed. 3 min equilibration was allowed after each GNP addition and before recording a measurement. Disposable cuvettes (DTS0012) were used for all measurements, with 5 measurements per run, with a 173° backscatter measuring angle and avalanche photodiode detector employed. Data presented is the mean of 5 measurements.

### Atomic Force Microscopy

Atomic Force Microscopy was performed on a Keysight N9418S 9500 Atomic Force Microscope in a tapping mode at room temperature (∼25 °C and 60% humidity) using Hi'Res-C15/Cr–Au probes (Apex Probes Ltd; 325 kHz resonance frequency, 40 N m^–1^ force constant). AFM images had a resolution of 512 × 512 px and were scanned with a speed of 1.5 lines per s. Surfaces were prepared by treating freshly cleaved mica with nickel(ii) chloride solution (40 μL, 100 mM; 20 min) washing with Milli-Q water and drying under nitrogen. Linearized pBR-322 plasmid DNA was prepared by incubation with Pst1-HF restriction enzyme (New England BioLabs) for 1 h at 37 °C, and purified using a QAIquick PCR purification column eluted with Milli-Q water. Its concentration was determined using a NanoDrop 8000 UV-vis spectrometer, and cleavage confirmed by Agarose Gel Electrophoresis.

23 μL of 6 nM GNP-lip-AA was added to an Eppendorf, followed by 153 μL of Milli-Q water. To this, 4 μL of 54.2 μM (in base pairs) linearized pBR-322 plasmid DNA was added and mixed before addition of 20 μL of 10× buffer (100 mM NaCl, 10 mM Tris-base). The final ratio was two NP's per linearized pBR-322 molecule (4361bp). DNA alone solution was prepared using 23 μL of Milli-Q water instead of the NP solution. Nanoparticle-DNA solution (40 μL) was pipetted onto the Nickel treated mica surface and left to incubate for 70 minutes. This was then washed with 500 μL of Milli-Q water and dried under nitrogen.

## Conclusions

We have shown that lipoic-gold nanoparticles (GNP) can be effective delivery platforms for Pt(ii) and Pt(iv) metallodrugs, transporting them into cells. The synthetic strategy we have employed allows rapid preparation of the GNP-drug conjugates, with enhanced loading of the drug on the surface of the nanocarriers, compared to other strategies reported so far with similar systems.¶ The drug is released in a pH-dependent fashion, with the highest level of release at lower pH. This is a potential advantage considering the acidic microenvironment that is associated with many tumor cells *in vivo*. The simple GNP-lip carrier particularly enhanced the efficacy of the platinum(iv) agents against lung A549 and ovarian A2780 tumor cells in culture.

Because the nanocarriers can also carry a fluorescent tag, these conjugates can also represent a system with a multimodal capability (drug delivery and imaging). The design has deliberately used a non-inert fluorescent tag that also plays a second (DNA-binding) role, though it should be possible in future designs to separate those functions into two molecular components allowing the fluorescence and cytotoxic activity to be separately modulated if desired. The nanoparticles and their conjugates, enter cells within 3 hours. They tend to cluster in vesicles (and this probably reflects their mechanism of uptake) but can escape and are seen in the cytoplasm, endoplasmic reticulum and nucleus.

Crucially, the introduction of the aminoanthraquinone (AA)-based fluorophore leads to enhanced toxicity of the gold nanocarrier, demonstrating that we can create nanocarriers that are is not just inert components, with a role just to deliver and release the drug; this opens new thinking in how a nanoparticle might be used not only as a drug carrier, and not only to carry both therapy and imaging agents, but to itself contribute to enhancing the cytotoxic action. The activity levels of the nanocarrier GNP-lip-AA were surprisingly high, and indeed may need to be modulated to suit the choice of drugs being delivered.

Excitingly we have shown that these AA functionalised nanoparticles (GNP-lip-AA) cause remarkable coiling/wrapping of DNA despite their anionic surface charge. This is an unexpected and very interesting new aspect of DNA binding since such an effect is more commonly associated with polycations. Given that cationic nanoparticles can disrupt membranes in the cell,^33^,^34^ anionic nanoparticles that can cause such dramatic effects on DNA may have fascinating potential for new approaches to in-cell nucleic acid recognition.

## Conflicts of interest

There are no conflicts to declare.

## Supplementary Material

Supplementary information

## References

[cit1] Johnstone T. C., Suntharalingam K., Lippard S. J. (2016). Chem. Rev..

[cit2] Maldonado C. R., Salassa L., Gomez-Blanco N., Mareque-Rivas J. C. (2013). Coord. Chem. Rev..

[cit3] Zhang L., Gu F. X., Chan J. M., Wang A. Z., Langer R. S., Farokhzad O. C. (2008). Clin. Pharmacol. Ther..

[cit4] Anniebell S., Gopinath S. C. B. (2018). Curr. Med. Chem..

[cit5] Min Y. Z., Mao C. Q., Xu D. C., Wang J., Liu Y. Z. (2010). Chem. Commun..

[cit6] Browning R. J., Reardon P. J. T., Parhizkar M., Pedley R. B., Edirisinghe M., Knowles J. C., Stride E. (2017). ACS Nano.

[cit7] Parker J. P., Ude Z., Marmion C. J. (2016). Metallomics.

[cit8] Duan X. P., He C. B., Kron S. J., Lin W. B. (2016). Wiley Interdiscip. Rev.: Nanomed. Nanobiotechnol..

[cit9] Bazak R., Houri M., El Achy S., Kamel S., Refaat T. (2015). J. Cancer Res. Clin. Oncol..

[cit10] Kim C. S., Duncan B., Creran B., Rotello V. M. (2013). Nano today.

[cit11] Ding H. M., Ma Y. Q. (2013). Sci. Rep..

[cit12] Riddell I. A. and Lippard S. J., in Metallo-Drugs: Development and Action of Anticancer Agents, ed. A. Sigel, H. Sigel, E. Freisinger and R. K. O. Sigel, De Gruyter, Berlin, 2018/02/03 edn, 2018, vol. 18, pp. 1–42.

[cit13] Johnstone T. C., Park G. Y., Lippard S. J. (2014). Anticancer Res..

[cit14] Reedijk J. (2009). Eur. J. Inorg. Chem..

[cit15] Hannon M. J. (2007). Pure Appl. Chem..

[cit16] Kenny R. G., Chuah S. W., Crawford A., Marmion C. J. (2017). Eur. J. Inorg. Chem..

[cit17] Venkatesh V. and Sadler P. J., in Metallo-Drugs: Development and Action of Anticancer Agents, ed. A. Sigel, H. Sigel, E. Freisinger and R. K. O. Sigel, De GruyterBerlin, 2018/02/03 edn, 2018, vol. 18, pp. 69–103.

[cit18] Olszewski U., Hamilton G. (2010). Anti Cancer Agents Med. Chem..

[cit19] Ruggiero E., Hernandez-Gil J., Mareque-Rivas J. C., Salassa L. (2015). Chem. Commun..

[cit20] Chin C. F., Yap S. Q., Li J., Pastorin G., Ang W. H. (2014). Chem. Sci..

[cit21] Song W., Li M., Tang Z., Li Q., Yang Y., Liu H., Duan T., Hong H., Chen X. (2012). Macromol. Biosci..

[cit22] Paraskar A., Soni S., Basu S., Amarasiriwardena C. J., Lupoli N., Srivats S., Roy R. S., Sengupta S. (2011). Nanotechnology.

[cit23] Kolishetti N., Dhar S., Valencia P. M., Lin L. Q., Karnik R., Lippard S. J., Langer R., Farokhzad O. C. (2010). Proc. Natl. Acad. Sci. U. S. A..

[cit24] Blanco N. G., Maldonado C. R., Mareque-Rivas J. C. (2009). Chem. Commun..

[cit25] Sztandera K., Gorzkiewicz M., Klajnert-Maculewicz B. (2019). Mol. Pharm..

[cit26] Adams S. J., Carrod A. J., Rochford L. A., Walker M., Pikramenou Z. (2018). ChemistrySelect.

[cit27] Rogers N. J., Jeffery H. C., Claire S., Lewis D. J., Zikeli G., Hodges N. J., Egginton S., Nash G. B., Pikramenou Z. (2017). Nanomedicine.

[cit28] Fratoddi I., Venditti I., Cametti C., Russo M. V. (2014). J. Mater. Chem. B.

[cit29] Yeh Y. C., Creran B., Rotello V. M. (2012). Nanoscale.

[cit30] King S. M., Claire S., Teixeira R. I., Dosumu A. N., Carrod A. J., Dehghani H., Hannon M. J., Ward A. D., Bicknell R., Botchway S. W., Hodges N. J., Pikramenou Z. (2018). J. Am. Chem. Soc..

[cit31] Lewis D. J., Pikramenou Z. (2014). Coord. Chem. Rev..

[cit32] Davies A., Lewis D. J., Watson S. P., Thomas S. G., Pikramenou Z. (2012). Proc. Natl. Acad. Sci. U. S. A..

[cit33] Castillo P. M., Jimenez-Ruiz A., Carnerero J. M., Prado-Gotor R. (2018). ChemPhysChem.

[cit34] Saha K., Bajaj A., Duncan B., Rotello V. M. (2011). Small.

[cit35] Sanchez-Paradinas S., Perez-Andres M., Almendral-Parra M. J., Rodriguez-Fernandez E., Milian A., Palacio F., Orfao A., Criado J. J., Fuentes M. (2014). J. Inorg. Biochem..

[cit36] Tsai D. H., Cho T. J., Elzey S. R., Gigault J. C., Hackley V. A. (2013). Nanoscale.

[cit37] Lee S. M., Tsai D. H., Hackley V. A., Brechbiel M. W., Cook R. F. (2013). Nanoscale.

[cit38] Craig G. E., Brown S. D., Lamprou D. A., Graham D., Wheate N. J. (2012). Inorg. Chem..

[cit39] Comenge J., Sotelo C., Romero F., Gallego O., Barnadas A., Parada T. G., Dominguez F., Puntes V. F. (2012). PLoS One.

[cit40] Comenge J., Romero F. M., Sotelo C., Dominguez F., Puntes V. (2010). J Control. Release.

[cit41] Brown S. D., Nativo P., Smith J. A., Stirling D., Edwards P. R., Venugopal B., Flint D. J., Plumb J. A., Graham D., Wheate N. J. (2010). J. Am. Chem. Soc..

[cit42] Patra C. R., Bhattacharya R., Mukherjee P. (2010). J. Med. Chem..

[cit43] England C. G., Miller M. C., Kuttan A., Trent J. O., Frieboes H. B. (2015). Eur. J. Pharm. Biopharm..

[cit44] Dhar S., Daniel W. L., Giljohann D. A., Mirkin C. A., Lippard S. J. (2009). J. Am. Chem. Soc..

[cit45] Shi Y., Goodisman J., Dabrowiak J. C. (2013). Inorg. Chem..

[cit46] Ren L., Chow G. M. (2003). Mater. Sci. Eng. C.

[cit47] Siemeling U., Bretthauer F., Bruhn C., Fellinger T. P., Tong W. L., Chan M. C. W. (2010). Z. Naturforschung B.

[cit48] Zhang S., Sun S. M., Zhou M. M., Wang L., Zhang B. (2017). Sci. Rep..

[cit49] Turcu I., Zarafu I., Popa M., Chifiriuc M. C., Bleotu C., Culita D., Ghica C., Ionita P. (2017). Nanomaterials.

[cit50] Langdon-Jones E. E., Pope S. J. A. (2014). Coord. Chem. Rev..

[cit51] Huang Q., Lu G., Shen H.-M., Chung M. C. M., Ong C. N. (2007). Med. Res. Rev..

[cit52] Alderden R. A., Mellor H. R., Modok S., Hambley T. W., Callaghan R. (2006). Biochem. Pharmacol..

[cit53] Bryce N. S., Zhang J. Z., Whan R. M., Yamamoto N., Hambley T. W. (2009). Chem. Commun..

[cit54] Jain P. K., Lee K. S., El-Sayed I. H., El-Sayed M. A. (2006). J. Phys. Chem. B.

[cit55] Goodman C. M., Chari N. S., Han G., Hong R., Ghosh P., Rotello V. M. (2006). Chem. Biol. Drug Des..

[cit56] Gearheart L. A., Ploehn H. J., Murphy C. J. (2001). J. Phys. Chem. B.

[cit57] Sandström P., Boncheva M., Åkerman B. (2003). Langmuir.

[cit58] Zhang X., Servos M. R., Liu J. (2012). Langmuir.

[cit59] Du Y., Xia L., Jo A., Davis R. M., Bissel P., Ehrich M. F., Kingston D. G. I. (2018). Bioconjug. Chem..

[cit60] Wang F., Wang Y.-C., Dou S., Xiong M.-H., Sun T.-M., Wang J. (2011). ACS Nano.

[cit61] Shabana A. M., Mondal U. K., Alam M. R., Spoon T., Ross C. A., Madesh M., Supuran C. T., Ilies M. A. (2018). ACS Appl. Mater. Interfaces.

[cit62] Emami F., Banstola A., Vatanara A., Lee S., Kim J. O., Jeong J.-H., Yook S. (2019). Mol. Pharm..

[cit63] Cui T., Liang J.-J., Chen H., Geng D.-D., Jiao L., Yang J.-Y., Qian H., Zhang C., Ding Y. (2017). ACS Appl. Mater. Interfaces.

[cit64] Fu Y., Feng Q., Chen Y., Shen Y., Su Q., Zhang Y., Zhou X., Cheng Y. (2016). Mol. Pharm..

[cit65] Wang G., Zhang J., Murray R. W. (2002). Anal. Chem..

[cit66] Biver T., Eltugral N., Pucci A., Ruggeri G., Schena A., Secco F., Venturini M. (2011). Dalton Trans..

[cit67] Hari K., Pichaimani A., Kumpati P. (2013). RSC Adv..

[cit68] Mehrabi M., Wilson R. (2007). Small.

[cit69] Grabar K. C., Freeman R. G., Hommer M. B., Natan M. J. (1995). Anal. Chem..

[cit70] Zhang L. T., Lu T. B., Gokel G. W., Kaifer A. E. (1993). Langmuir.

[cit71] Lin S. Y., Tsai Y. T., Chen C. C., Lin C. M., Chen C. H. (2004). J. Phys. Chem. B.

[cit72] Hannon M. J., Moreno V., Prieto M. J., Molderheim E., Sletten E., Meistermann I., Isaac C. J., Sanders K. J., Rodger A. (2001). Angew. Chem., Int. Ed..

[cit73] Carnerero J. M., Masuoka S., Baba H., Yoshikawa Y., Prado-Gotor R., Yoshikawa K. (2018). RSC Adv..

[cit74] Carnerero J. M., Jimenez-Ruiz A., Grueso E. M., Prado-Gotor R. (2017). Phys. Chem. Chem. Phys..

[cit75] Zinchenko A., Yoshikawa K. (2015). Curr. Opin. Colloid Interface Sci..

