# Supplementary material for: Assisted delivery of anti-tumour platinum drugs using DNA-coiling gold nanoparticles bearing lumophores and intercalators: towards a new generation of multimodal nanocarriers with enhanced action
Source: Chem Sci. 2019 Aug 13;10(40):9244–56. doi: 10.1039/c9sc02640a (PMC7003971; doi:10.1039/c9sc02640a)
Supplement: Supplementary file 1 [file SC-010-C9SC02640A-s001.pdf]

SUPPLEMENTARY INFORMATION

Assisted delivery of anti-tumour platinum drugs using DNA-coiling gold nanoparticles bearing lumophores and intercalators: Towards a new generation of multimodal nanocarriers with enhanced action.

Ana B. Caballero,[a] Lucia Cardo,[a] Sunil Claire,[a] James S. Craig,[b] Anton Vladyka,[a] Tim Albrecht,[a] Luke A. Rochford,[a] Nikolas J. Hodges,[c] Zoe Pikramenou *[a] and Michael J. Hannon *[a]

(a) School of Chemistry; (b) Physical Sciences for Health Centre (c) School of Biosciences, University of Birmingham, Edgbaston, Birmingham B15 2TT, United Kingdom.

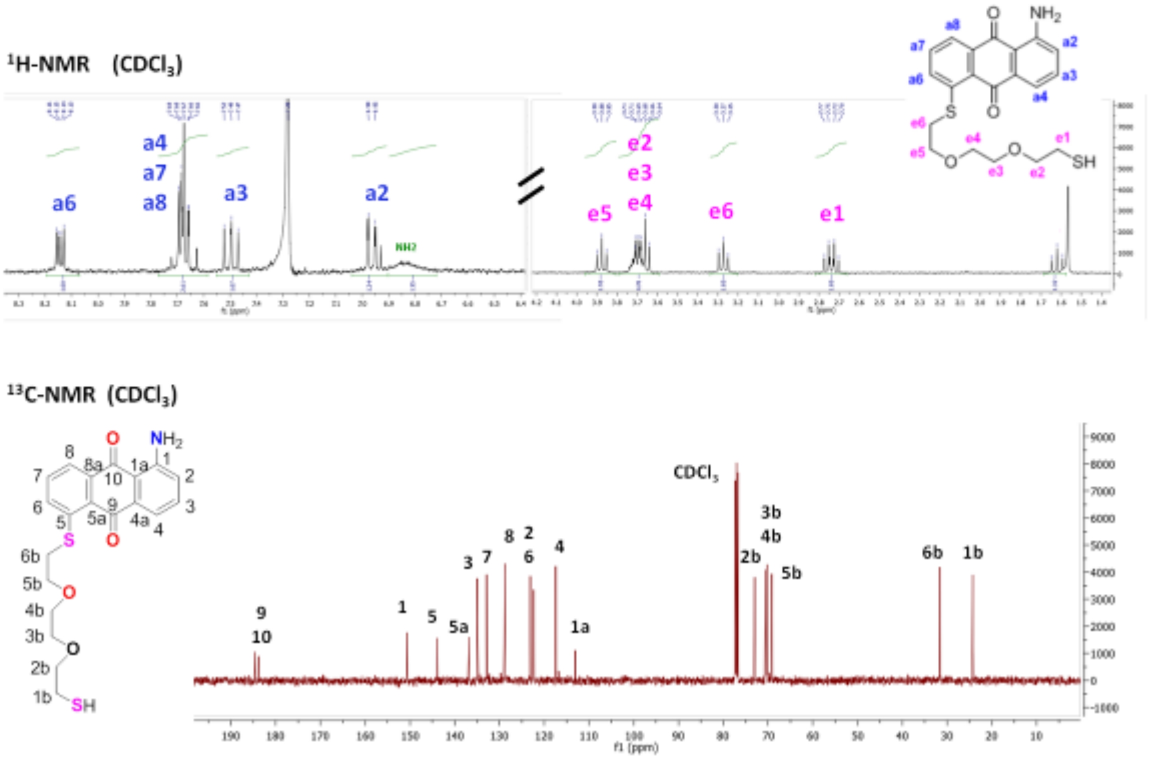

Figure S1. ^{1}H-NMR and ^{13}C-NMR spectra of *AA* compound.

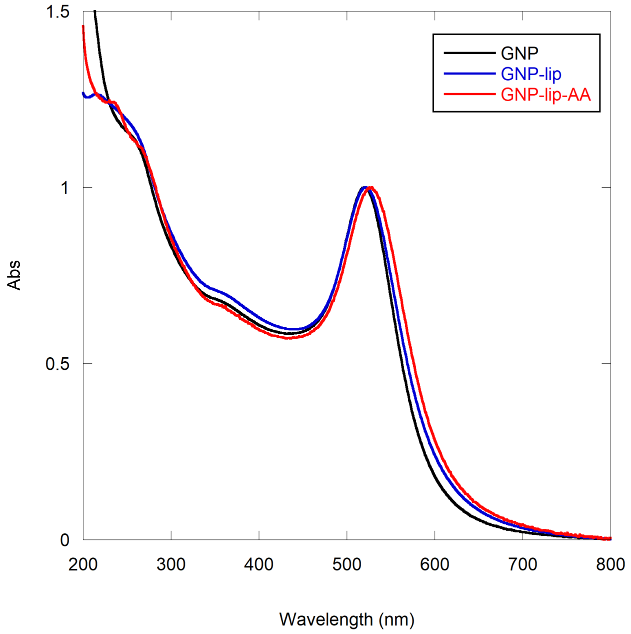

Figure S2. Normalized UV-Vis spectra of *GNP*, *GNP-lip* and *GNP-lip-AA* in water. The magnitude of the red shift is greater than instrumentational error

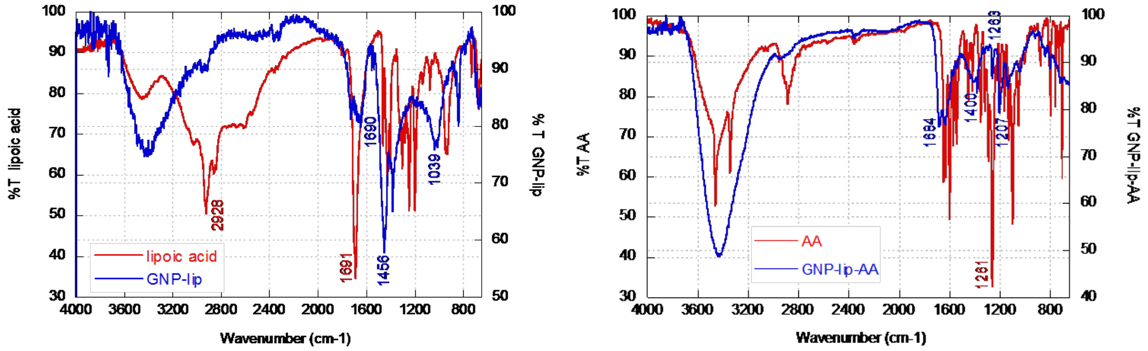

Figure S3. FT-IR spectra of (a) *GNP-lip* and (b) *GNP-lip-AA*. The disappearance of the S-H band in the nanoparticles confirms the binding to the gold surface via this group.

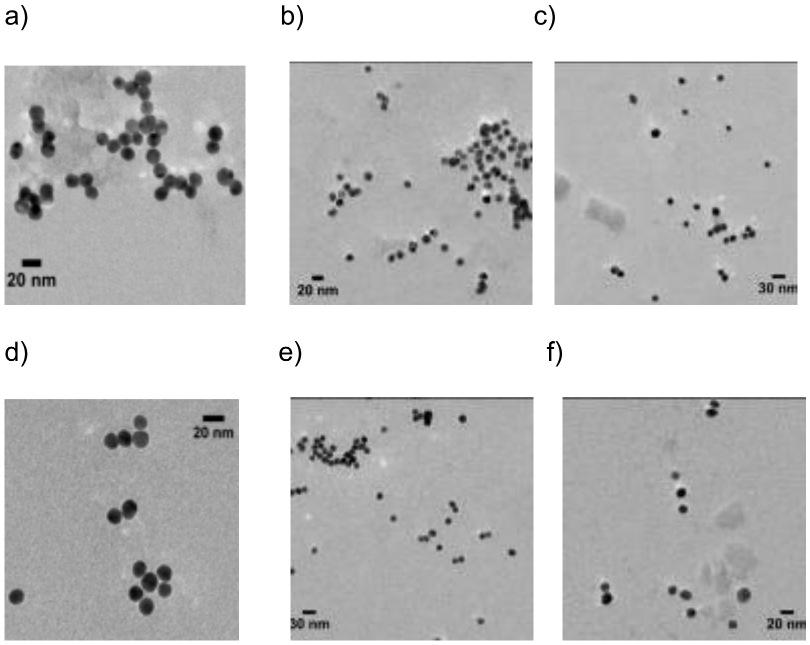

Figure S4. TEM images of (a) *GNP-lip*, (b) *GNP-lip-PtII*, (c) *GNP-lip-PtIVsuc*, (d) *GNP-lip-AA*, (e) *GNP-lip-AA-PtII,* (f) *GNP-lip-AA-PtIVsuc*.

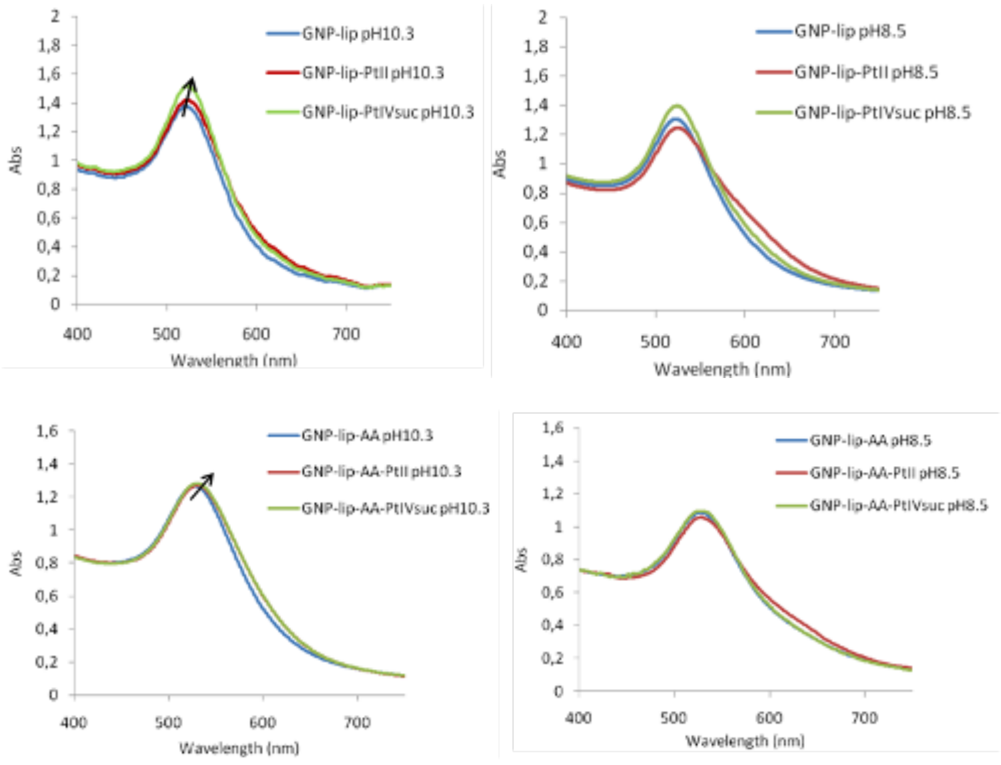

r

Figure S5. UV-Vis spectra of the nanocarriers *GNP-Lip* (top) and *GNP-Lip-AA* (bottom) before and after functionalization with platinum drugs and before and after purification (on the left and on the right, respectively).

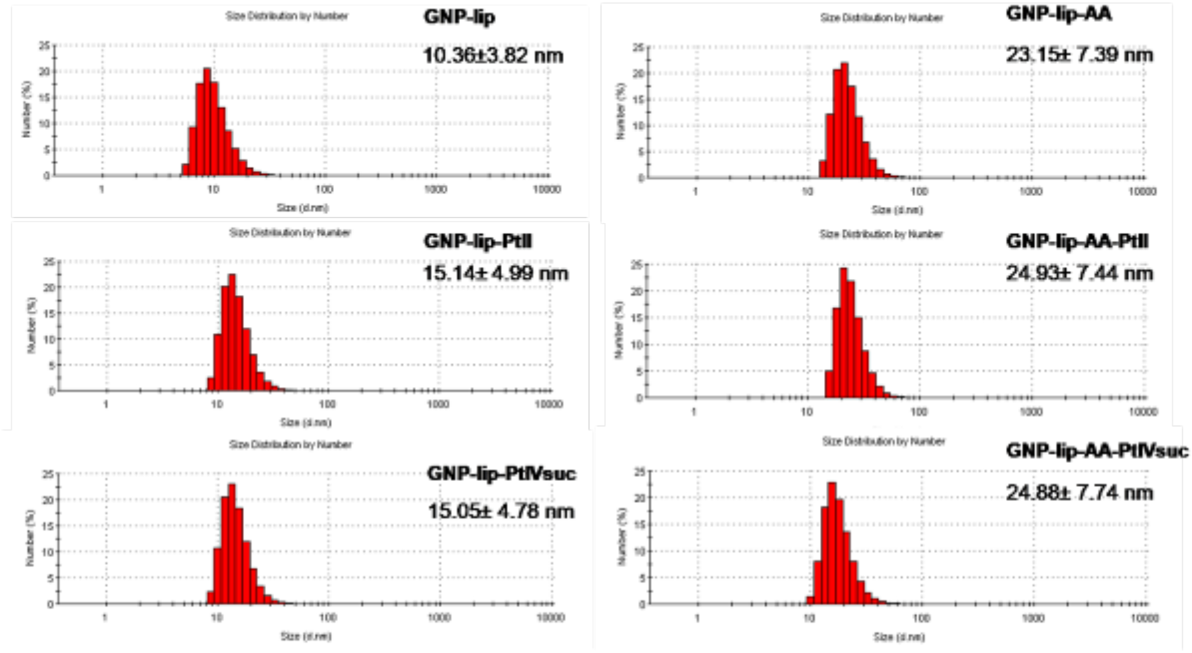

Figure S6. Dynamic light scattering (DLS) particle size distribution graphs of the conjugates. Addition of platinum does not affect dispersity.

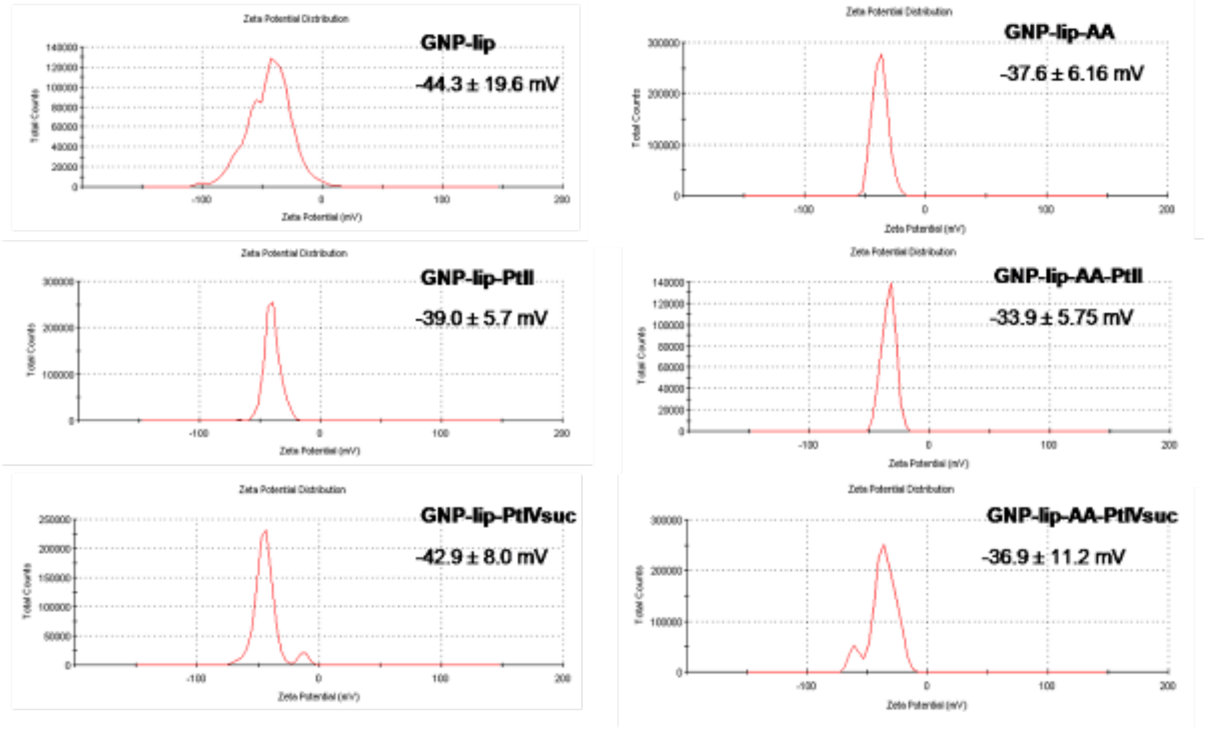

Figure S7. ξ- potential distribution graphs of the conjugates.

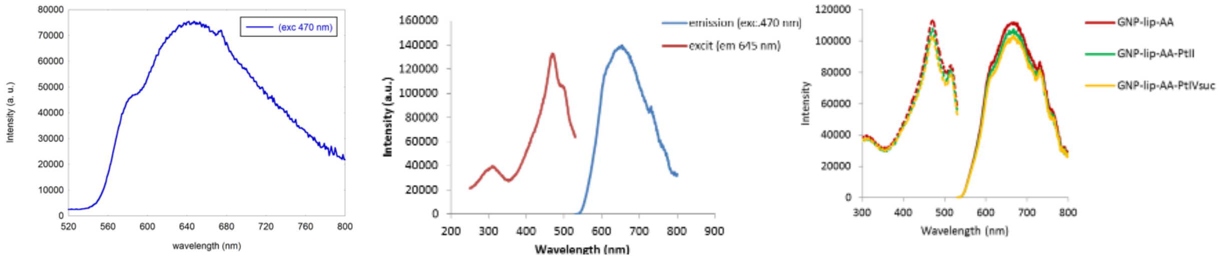

Figure S8. On the left, emission of *AA* upon excitation at 470 nm. *AA* is poorly soluble in water, therefore it was pre-dissolved in ethanol and added to water to get a final concentration of 2 μM. In the middle, excitation (red line) and emission spectra (blue line) of *GNP-lip-AA* in water. On the right, excitation (dashed lines) and emission spectra (solid lines) of *GNP-lip-AA* before and after binding of *PtII* and *PtIVsuc* in water. All measurements were carried in a Edinburgh Instruments FLSP920 fluorescence spectrometer using a 1 mL quartz cuvette.

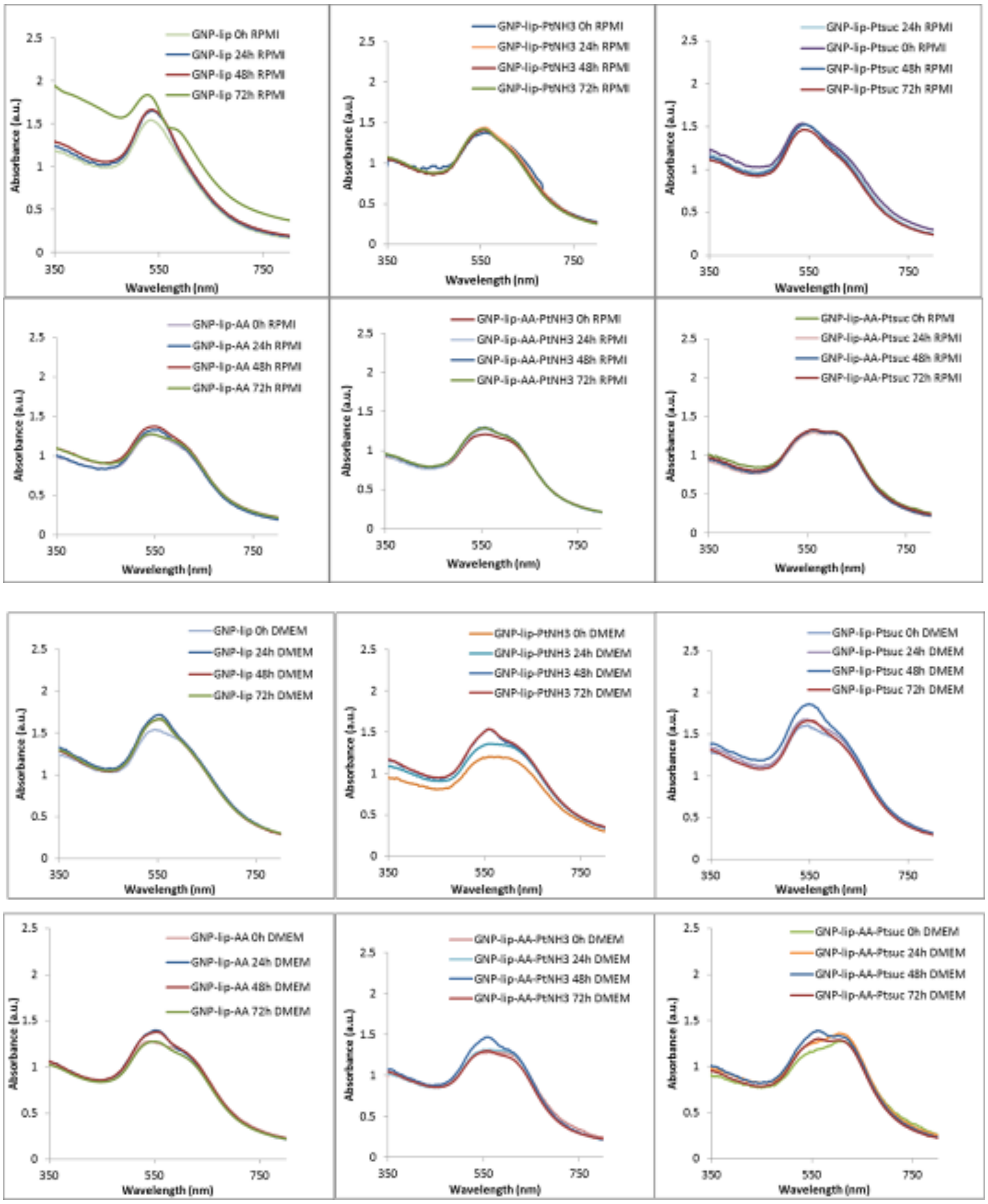

Figure S9. UV-Vis spectra of Pt drug-nanocarriers in supplemented cell media RPMI 1640 (top) and DMEM (bottom) at different times.

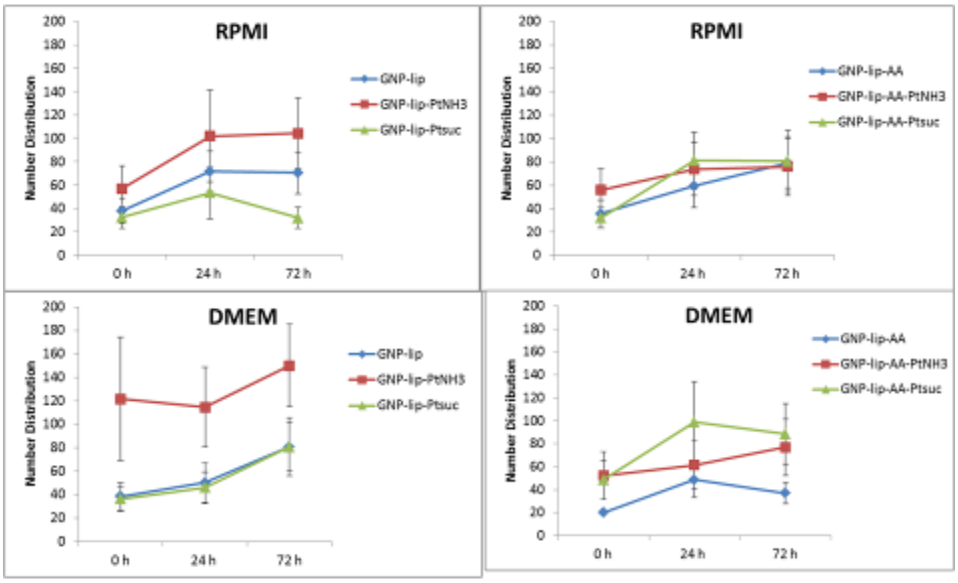

Figure S10. DLS particle size measurements in cell media at different times.

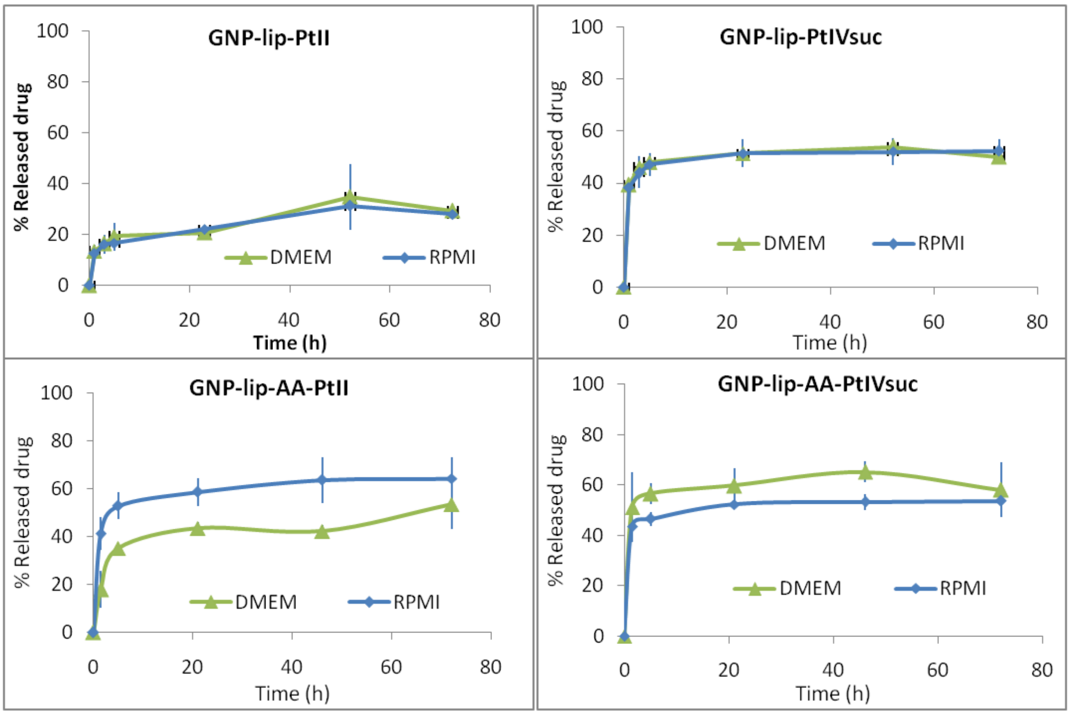

Figure S11. *In vitro* cumulative release of Pt drug from their respective conjugates in RPMI 1640 and DMEM cell media (37 ºC, 5% CO_2, pH 7.4).

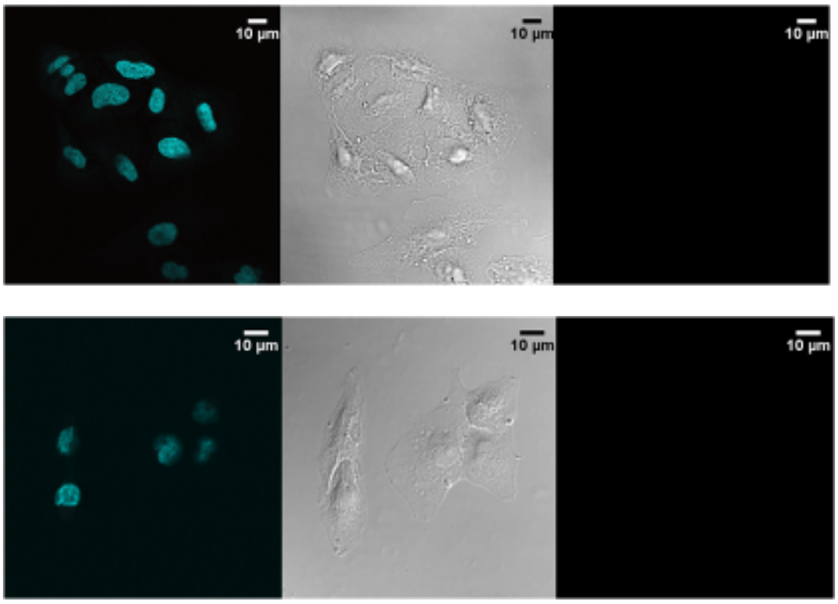

Figure S12. CLSM images of non-treated A549 cells after 3 h (top) and 24 h (bottom). From left to right, fluorescence overlay image of AuNP fluorescence (red) and Hoechst nuclear stain (cyan), bright field mode, and reflection mode.

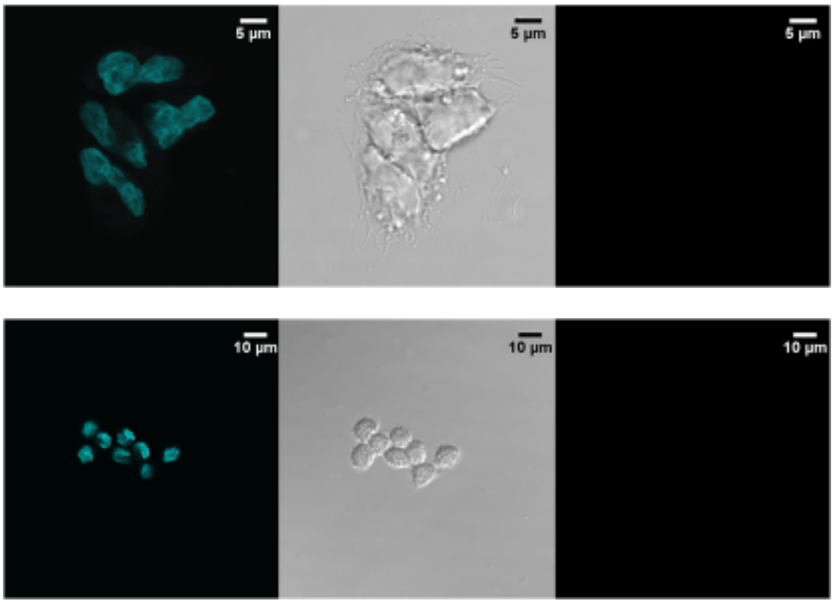

Figure S13. CLSM images of non-treated A2780 cells after 3 h (top) and 24 h (bottom). From left to right, fluorescence overlay image of AuNP fluorescence (red) and Hoechst nuclear stain (cyan), bright field mode, and reflection mode.

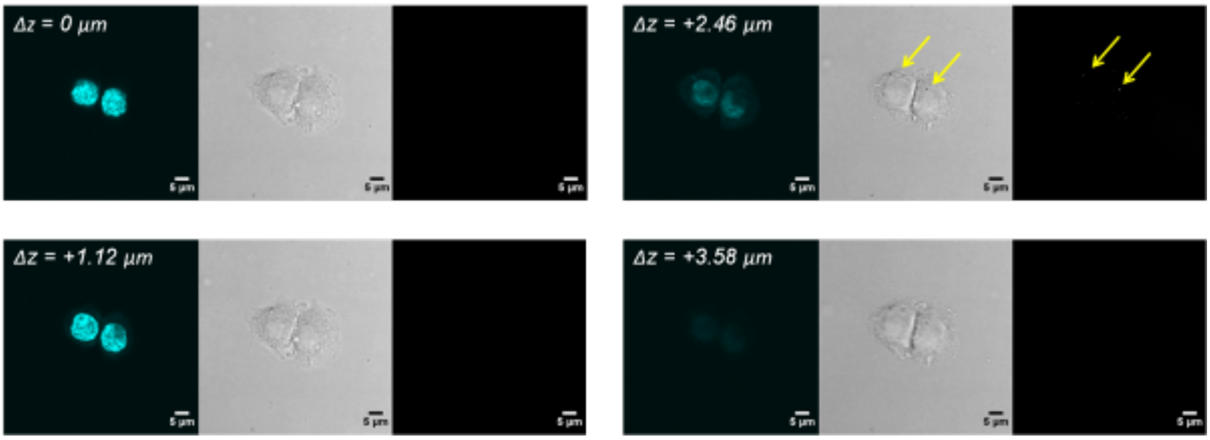

Figure S14. CLSM *z*-stack images of A549 cells treated with **GNP-lip** for 3 h. From left to right, fluorescence overlay image of AuNP fluorescence (red) and Hoechst nuclear stain (cyan), bright field mode, and reflection mode..

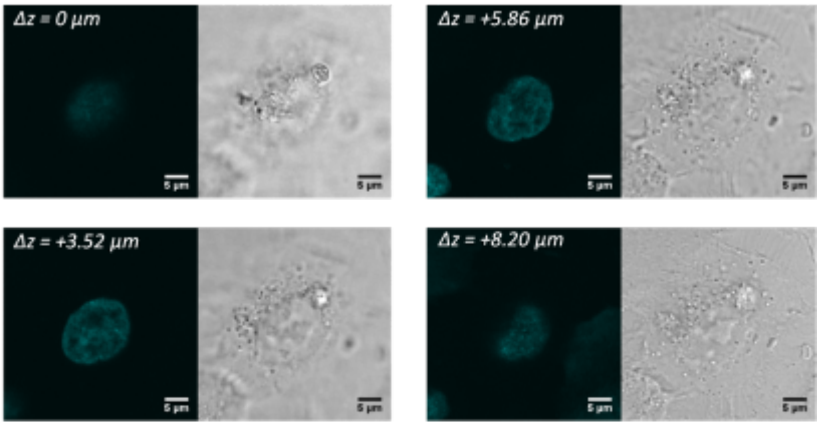

Figure S15. CLSM *z*-stack images of A549 cells treated with **GNP-lip** for 24 h. From left to right, fluorescence overlay image of AuNP fluorescence (red) and Hoechst nuclear stain (cyan), and bright field mode.

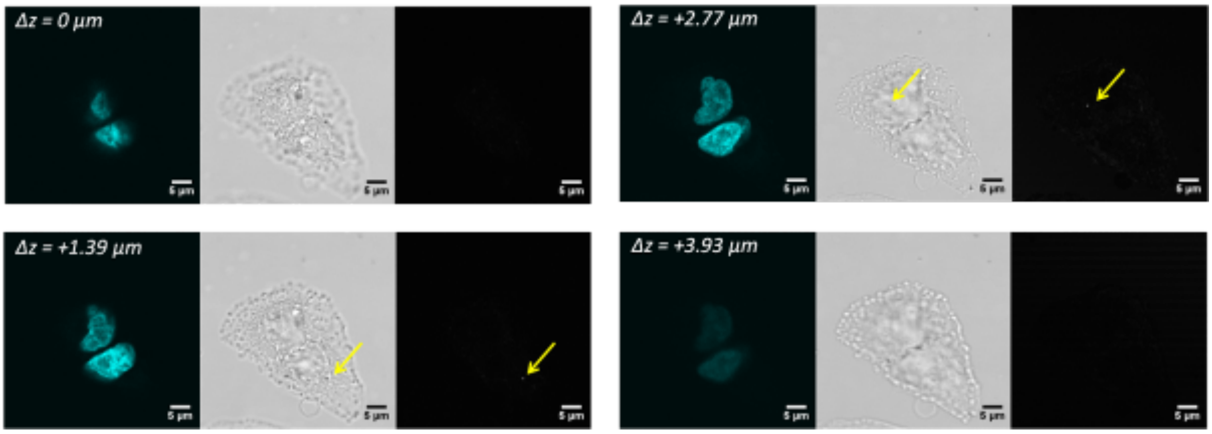

Figure S16. CLSM *z*-stack images of A549 cells treated with **GNP-lip-PtII** for 3 h. From left to right, fluorescence overlay image of AuNP fluorescence (red) and Hoechst nuclear stain (cyan), and bright field mode.

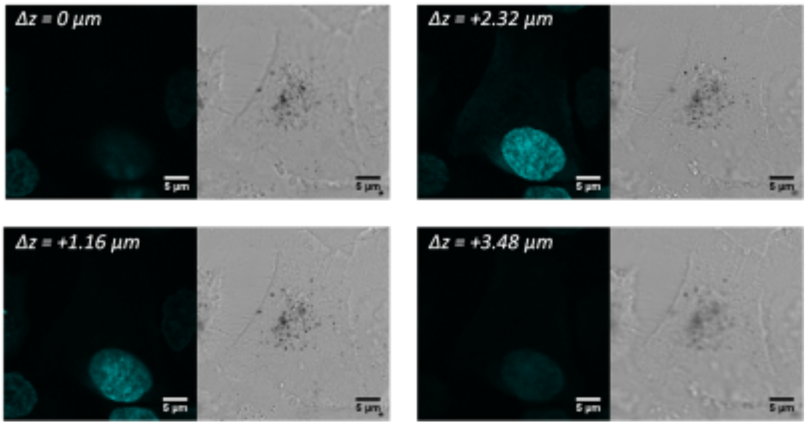

Figure S17. CLSM *z*-stack images of A549 cells treated with **GNP-lip-PtII** for 24 h. From left to right, fluorescence overlay image of AuNP fluorescence (red) and Hoechst nuclear stain (cyan), and bright field mode.

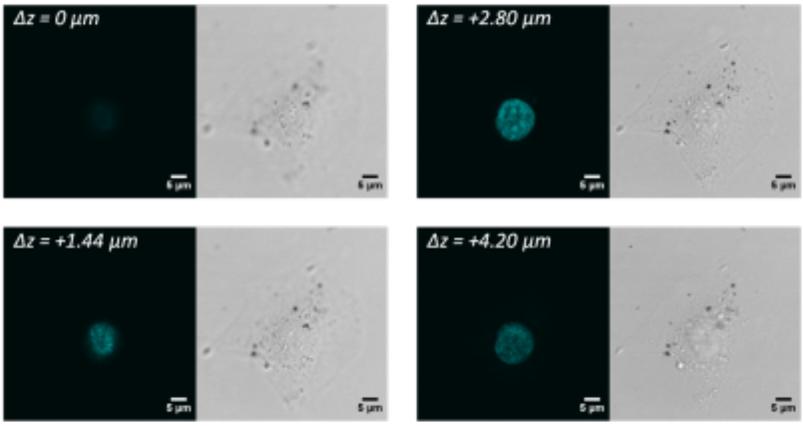

Figure S18. CLSM *z*-stack images of A549 cells treated with **GNP-lip-PtIVsuc** for 3 h. From left to right, fluorescence overlay image of AuNP fluorescence (red) and Hoechst nuclear stain (cyan), and bright field mode.

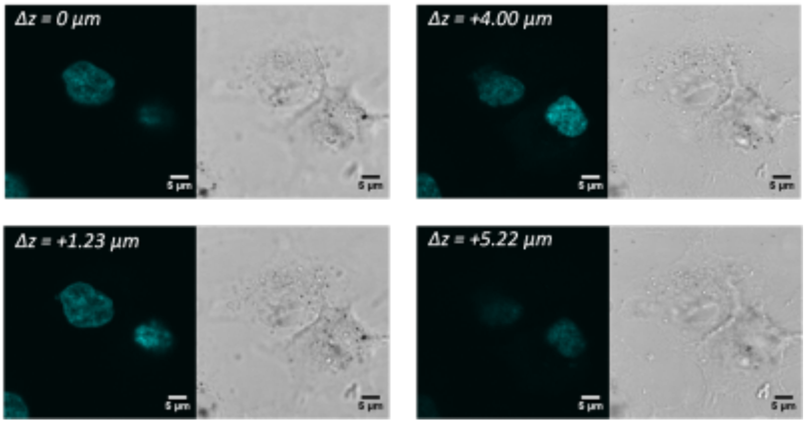

Figure S19. CLSM *z*-stack images of A549 cells treated with **GNP-lip-PtIVsuc** for 24 h. From left to right, fluorescence overlay image of AuNP fluorescence (red) and Hoechst nuclear stain (cyan), and bright field mode.

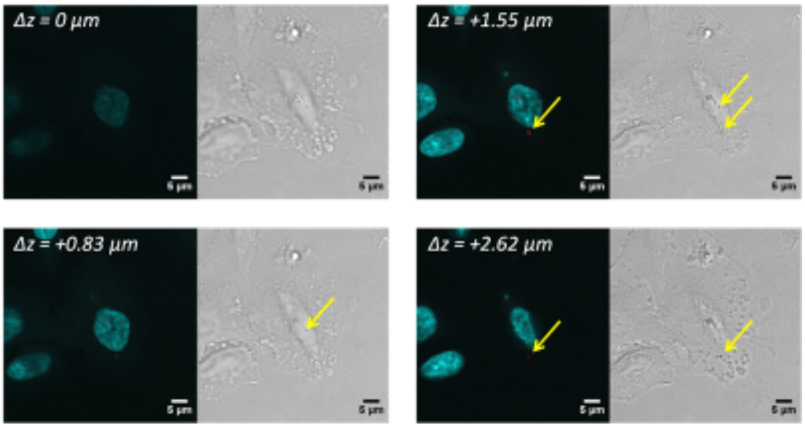

Figure S20. CLSM *z*-stack images of A549 cells treated with **GNP-lip-AA** for 3 h. From left to right, fluorescence overlay image of AuNP fluorescence (red) and Hoechst nuclear stain (cyan), and bright field mode.

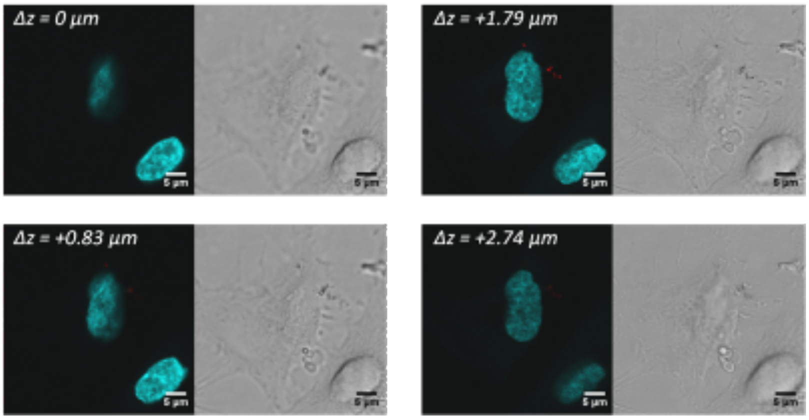

Figure S21. CLSM *z*-stack images of A549 cells treated with **GNP-lip-AA** for 24 h. From left to right, fluorescence overlay image of AuNP fluorescence (red) and Hoechst nuclear stain (cyan), and bright field mode.

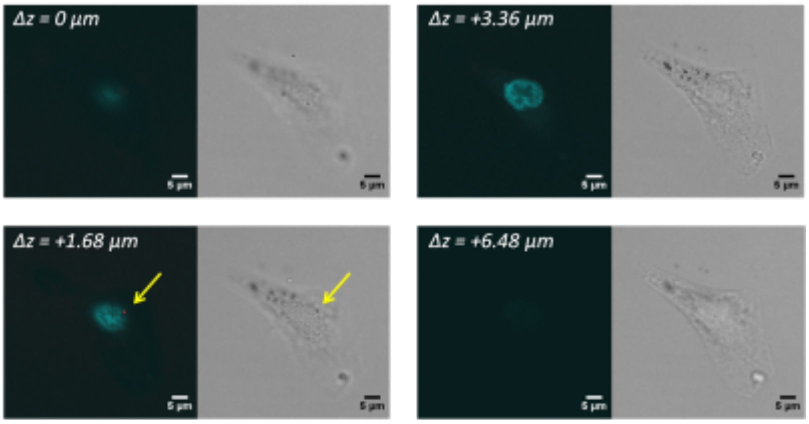

Figure S22. CLSM *z*-stack images of A549 cells treated with **GNP-lip-AA-PtII** for 3 h. From left to right, fluorescence overlay image of AuNP fluorescence (red) and Hoechst nuclear stain (cyan), and bright field mode.

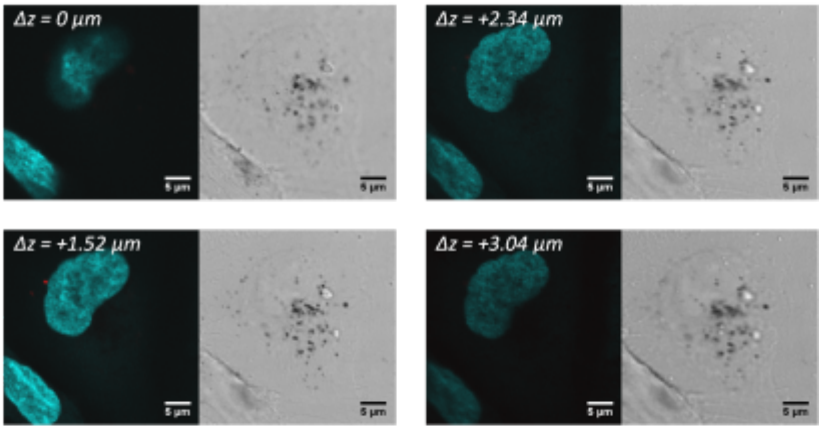

Figure S23. CLSM *z*-stack images of A549 cells treated with **GNP-lip-AA-PtII** for 24 h. From left to right, fluorescence overlay image of AuNP fluorescence (red) and Hoechst nuclear stain (cyan), and bright field mode.

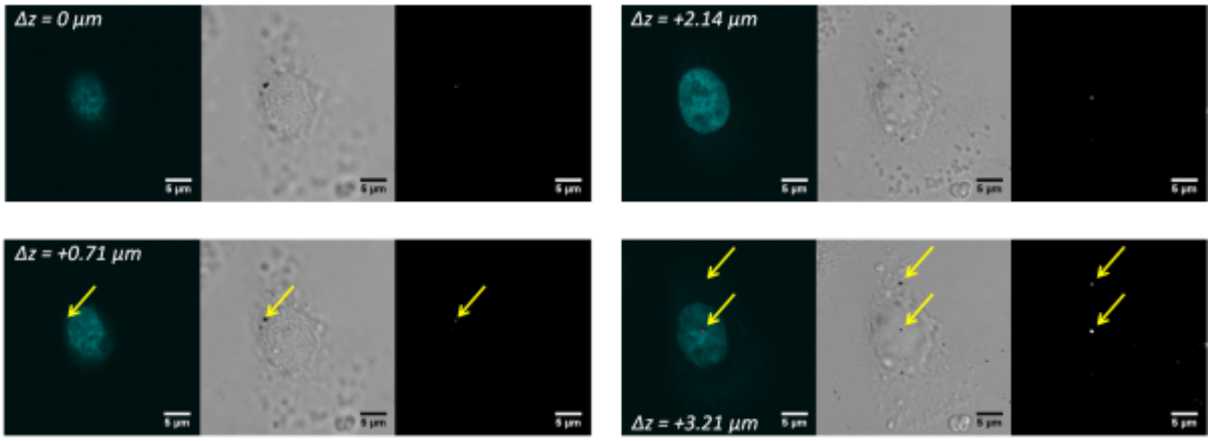

Figure S24. CLSM *z*-stack images of A549 cells treated with **GNP-lip-AA-PtIVsuc** for 3 h. From left to right, fluorescence overlay image of AuNP fluorescence (red) and Hoechst nuclear stain (cyan), bright field mode, and reflection mode.

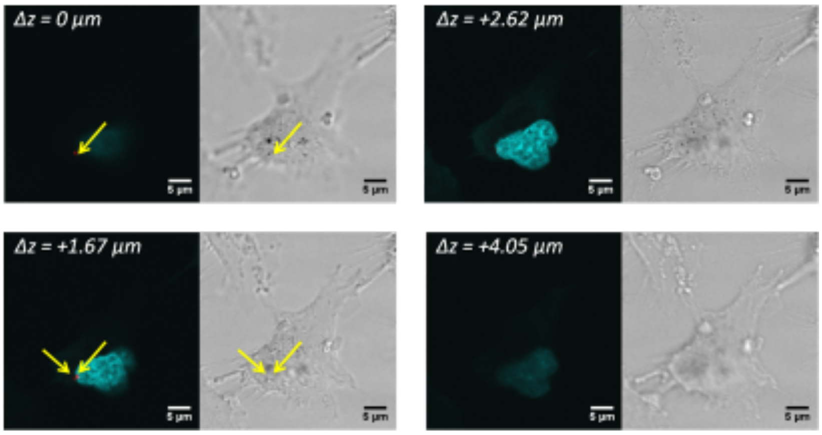

Figure S25. CLSM *z*-stack images of A549 cells treated with **GNP-lip-AA-PtIVsuc** for 24 h. From left to right, fluorescence overlay image of AuNP fluorescence (red) and Hoechst nuclear stain (cyan), and bright field mode.

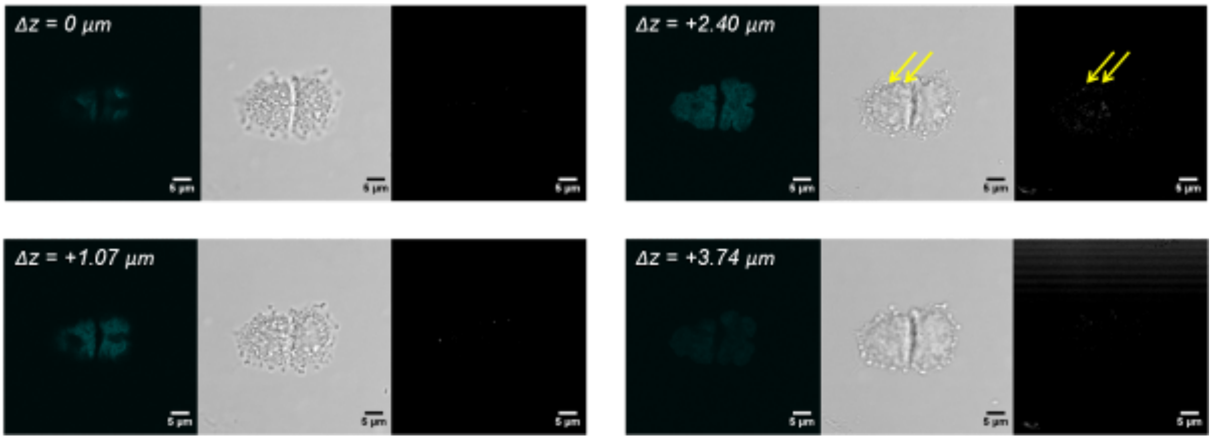

Figure S26. CLSM *z*-stack images of A2780 cells treated with **GNP-lip** for 3 h. From left to right, fluorescence overlay image of AuNP fluorescence (red) and Hoechst nuclear stain (cyan), bright field mode, and reflection mode.

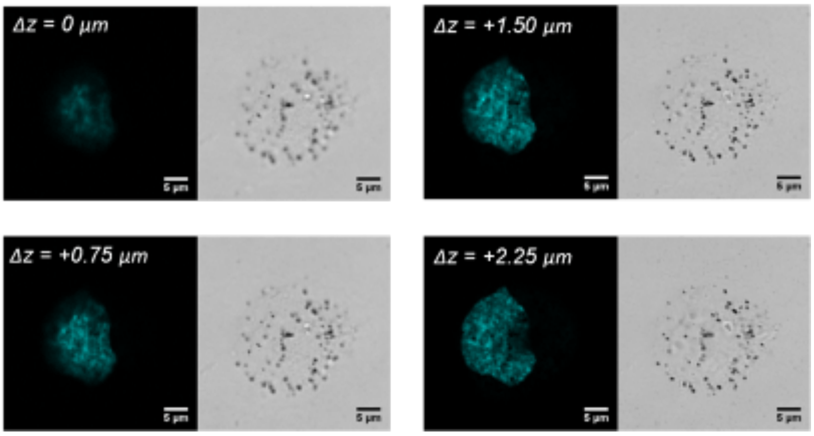

Figure S27. CLSM *z*-stack images of A2780 cells treated with **GNP-lip** for 24 h. From left to right, fluorescence overlay image of AuNP fluorescence (red) and Hoechst nuclear stain (cyan), and bright field mode.

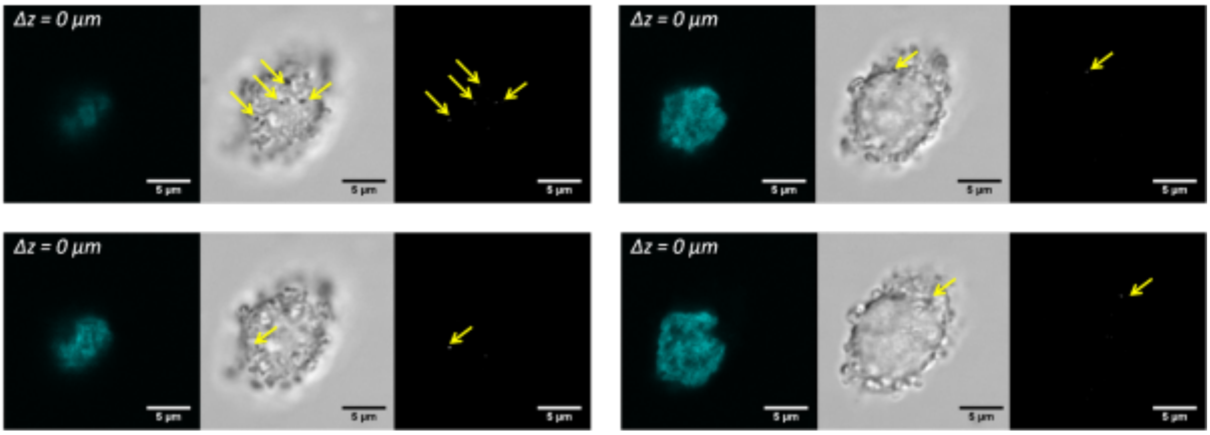

Figure S28. CLSM *z*-stack images of A2780 cells treated with **GNP-lip-PtII** for 3 h. From left to right, fluorescence overlay image of AuNP fluorescence (red) and Hoechst nuclear stain (cyan), bright field mode and reflection mode.

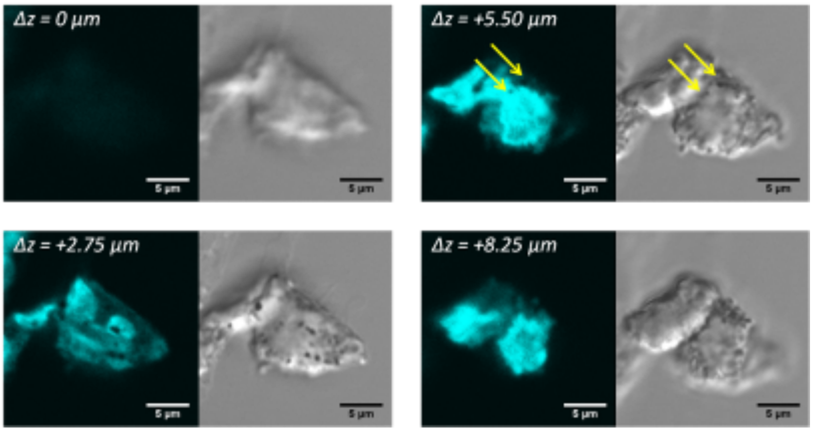

Figure S29. CLSM *z*-stack images of A2780 cells treated with **GNP-lip-PtII** for 24 h. From left to right, fluorescence overlay image of AuNP fluorescence (red) and Hoechst nuclear stain (cyan), and bright field mode.

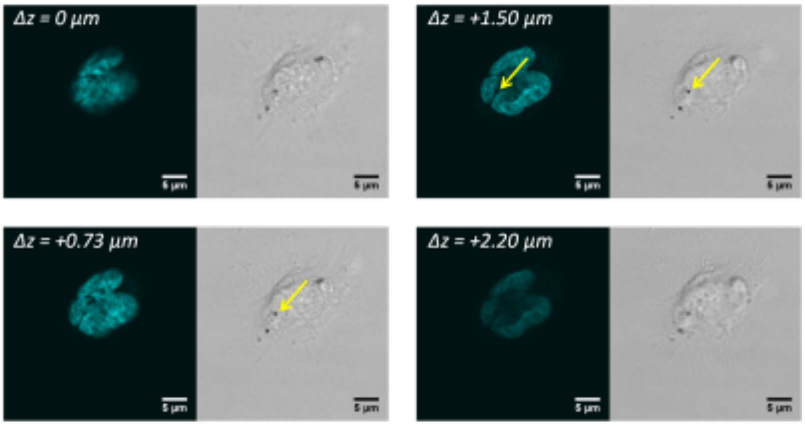

Figure S30. CLSM *z*-stack images of A2780 cells treated with **GNP-lip-PtIVsuc** for 3 h. From left to right, fluorescence overlay image of AuNP fluorescence (red) and Hoechst nuclear stain (cyan), and bright field mode.

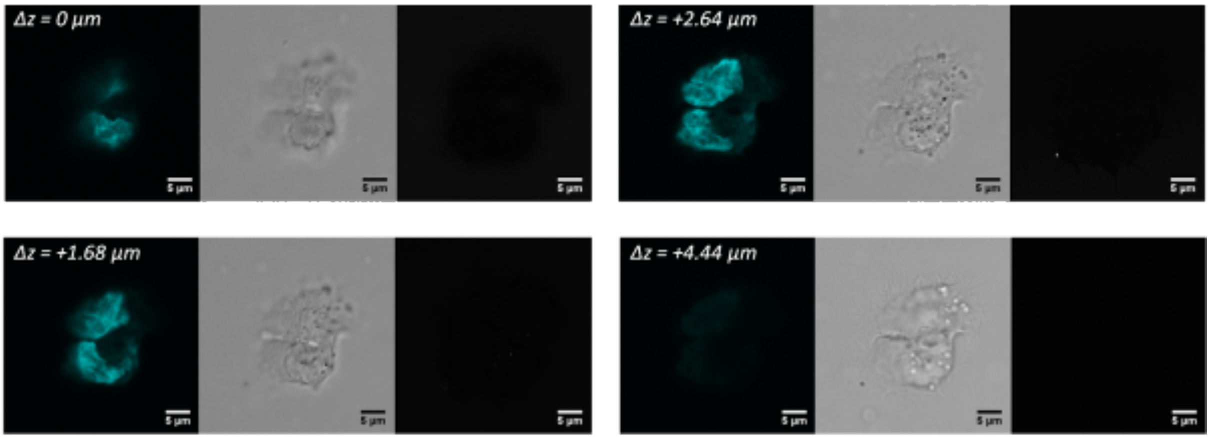

Figure S31. CLSM *z*-stack images of A2780 cells treated with **GNP-lip-PtIVsuc** for 24 h. From left to right, fluorescence overlay image of AuNP fluorescence (red) and Hoechst nuclear stain (cyan), bright field mode, and reflection mode.

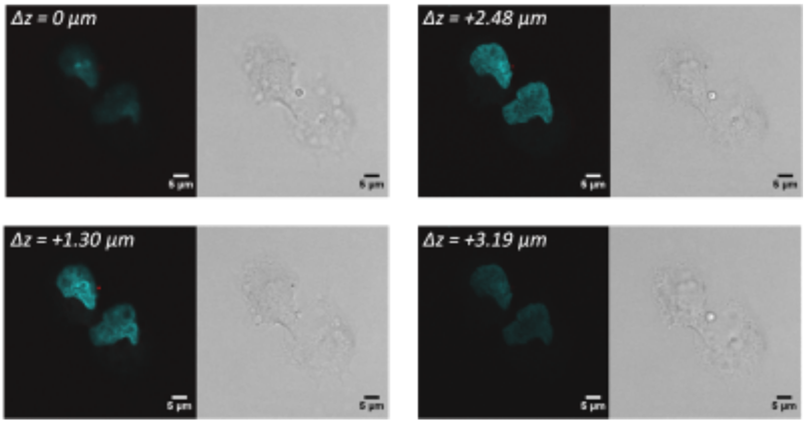

Figure S32. CLSM *z*-stack images of A2780 cells treated with **GNP-lip-AA** for 3 h. From left to right, fluorescence overlay image of AuNP fluorescence (red) and Hoechst nuclear stain (cyan), and bright field mode.

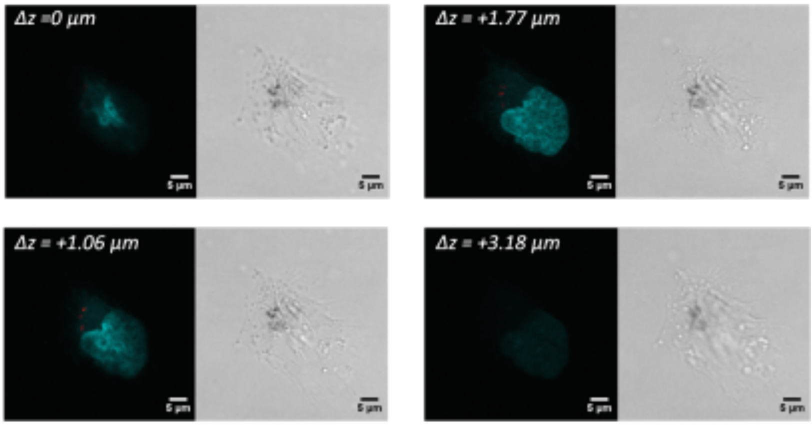

Figure S33. CLSM *z*-stack images of A2780 cells treated with **GNP-lip-AA** for 24 h. From left to right, fluorescence overlay image of AuNP fluorescence (red) and Hoechst nuclear stain (cyan), and bright field mode.

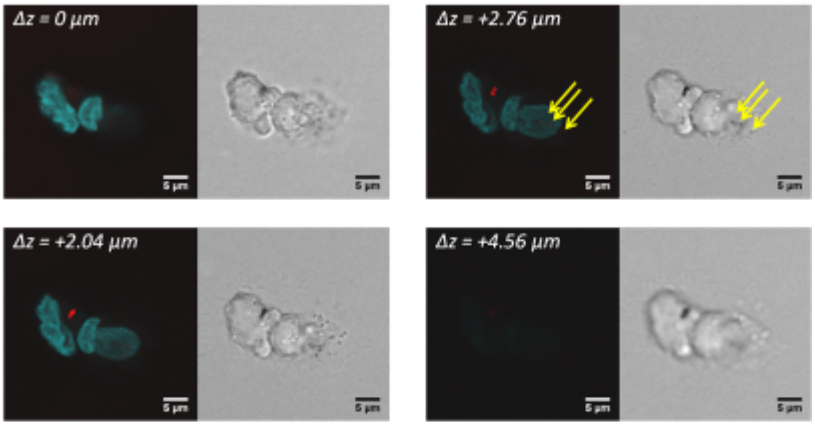

Figure S34. CLSM *z*-stack images of A2780 cells treated with **GNP-lip-AA-PtII** for 3 h. From left to right, fluorescence overlay image of AuNP fluorescence (red) and Hoechst nuclear stain (cyan), and bright field mode.

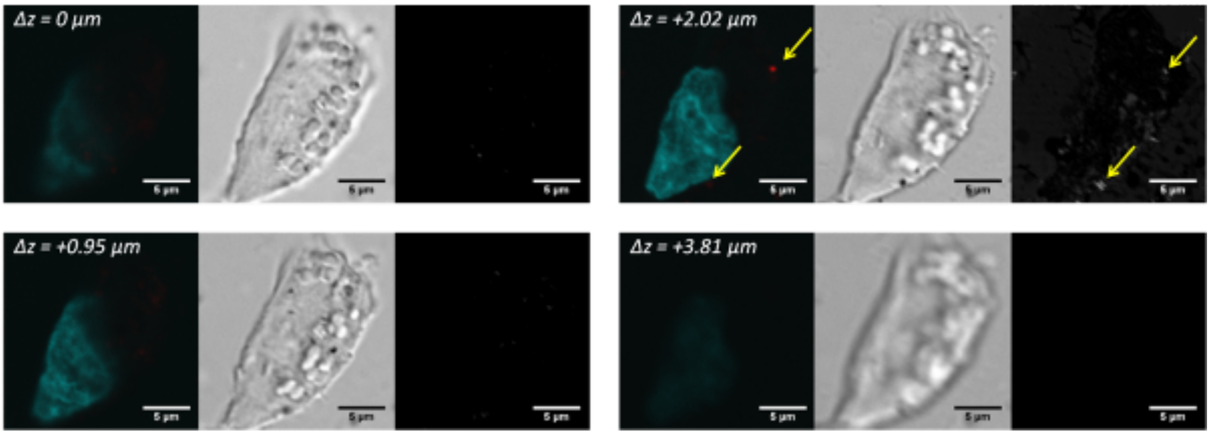

Figure S35. CLSM *z*-stack images of A2780 cells treated with **GNP-lip-AA-PtII** for 24 h. From left to right, fluorescence overlay image of AuNP fluorescence (red) and Hoechst nuclear stain (cyan), bright field mode, and reflection mode.

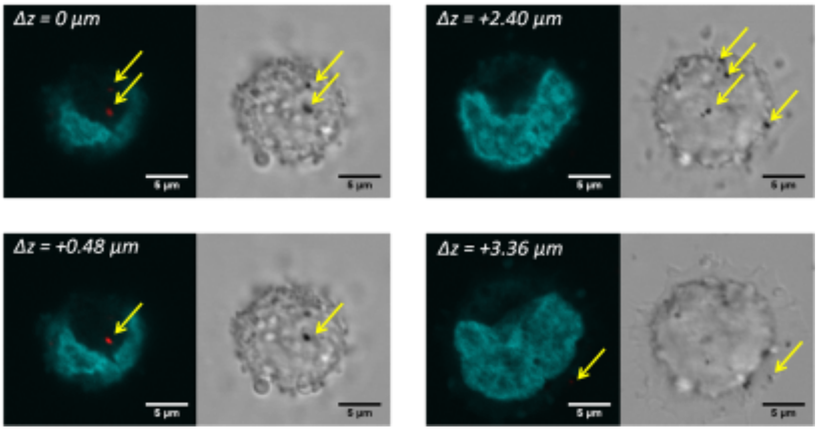

Figure S36. CLSM *z*-stack images of A2780 cells treated with **GNP-lip-AA-PtIVsuc** for 3 h. From left to right, fluorescence overlay image of AuNP fluorescence (red) and Hoechst nuclear stain (cyan), and bright field mode.

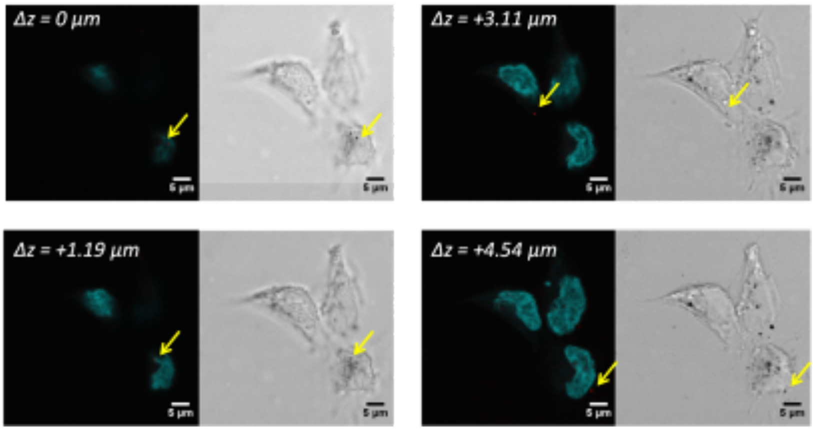

Figure S37. CLSM *z*-stack images of A2780 cells treated with **GNP-lip-AA-PtIVsuc** for 24 h. From left to right, fluorescence overlay image of AuNP fluorescence (red) and Hoechst nuclear stain (cyan), and bright field mode.

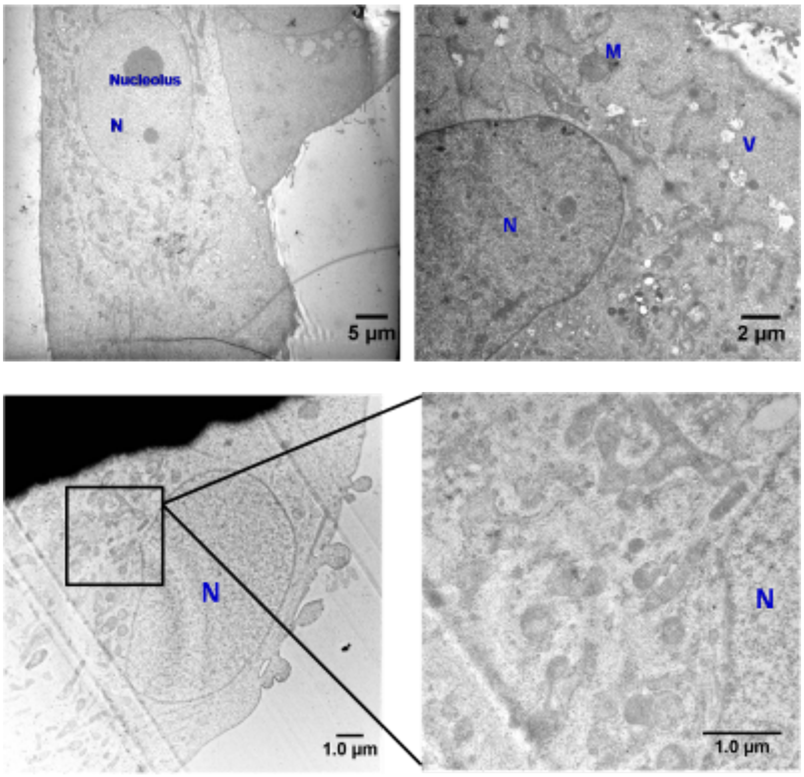

Figure S38. TEM images untreated A549 (top) and A2780 cells (bottom). N stands for nucleus, M for mitochondria, and V for vacuole.

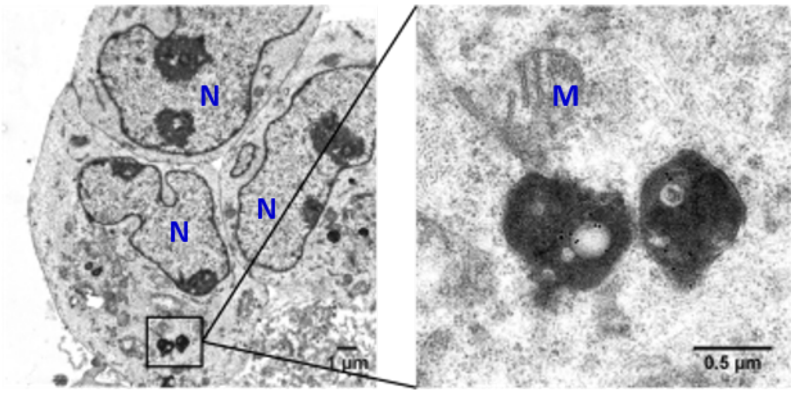

Figure S39. TEM images of A2780 cells treated with **GNP-lip** for 24 h. N: nucleus, M: mitochondria.

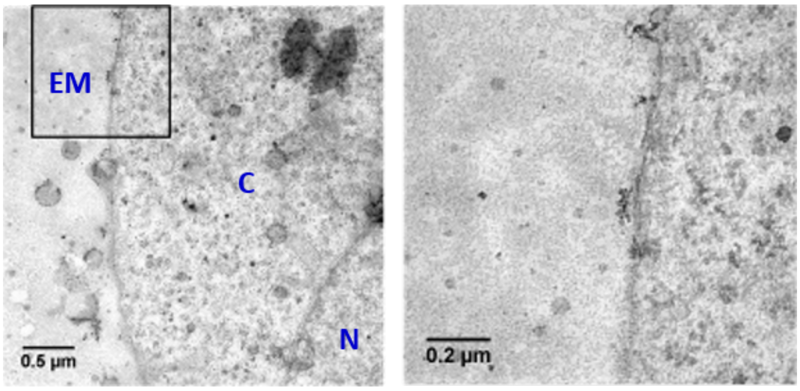

Figure S40. TEM images of A2780 cells treated with **GNP-lip-PtII** for 24 h. EM: extracellular media, C: cytoplasm; N: nucleus.

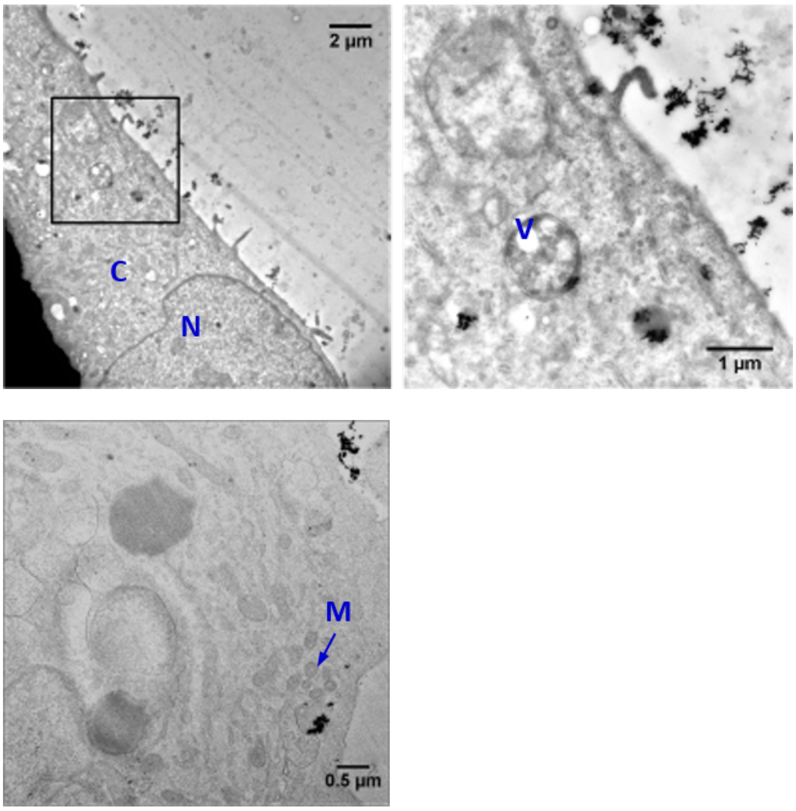

Figure S41. TEM images of A549 (top) and A2780 cells (bottom) treated with **GNP-lip-PtIVsuc** for 24 h. C: cytoplasm, N: nucleus, M: mitochondrion, V: vacuole.

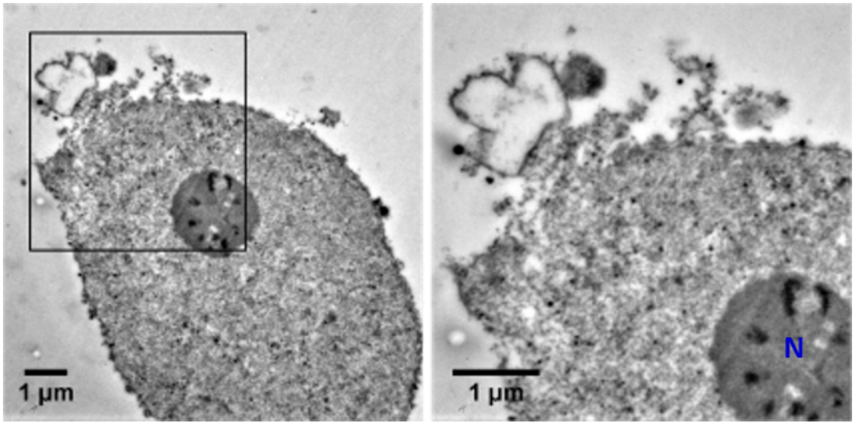

Figure S42. TEM images of A2780 cells treated with **GNP-lip-AA** for 24 h. N: nucleus.

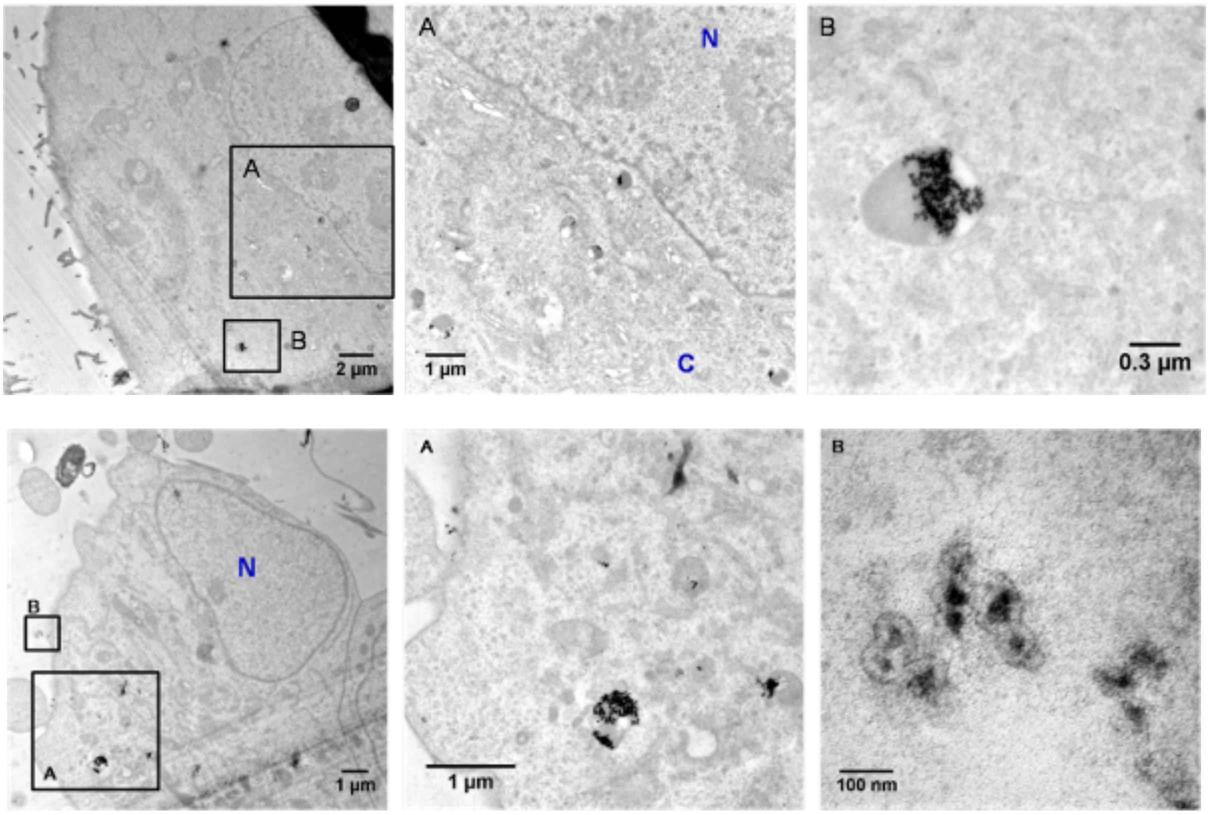

Figure S43. TEM images of A549 (top) and A2780 cells (bottom) treated with **GNP-lip-AA-PtII** for 24 h. N: nucleus, C: cytoplasm.

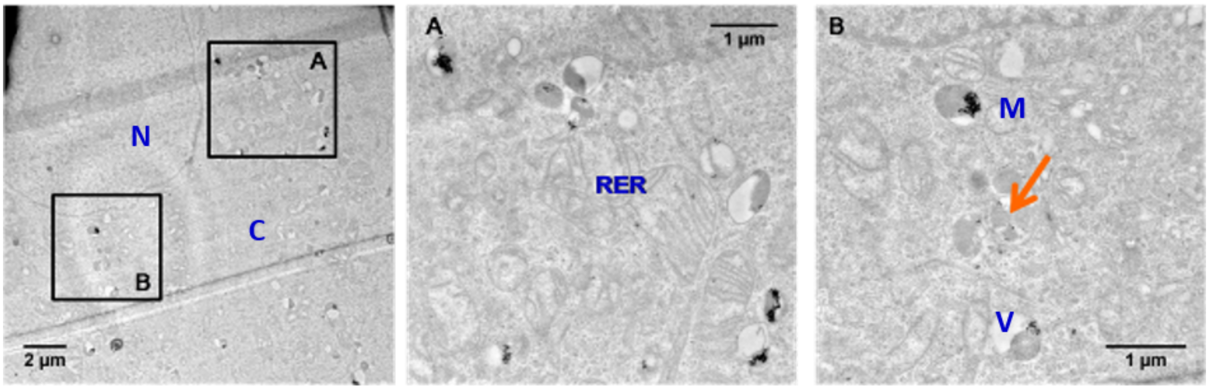

Figure S44. TEM images of A549 treated with **GNP-lip-AA-PtIVsuc** for 24 h. RER: rough endoplasmic reticulum, N: nucleus, C: cytoplasm.

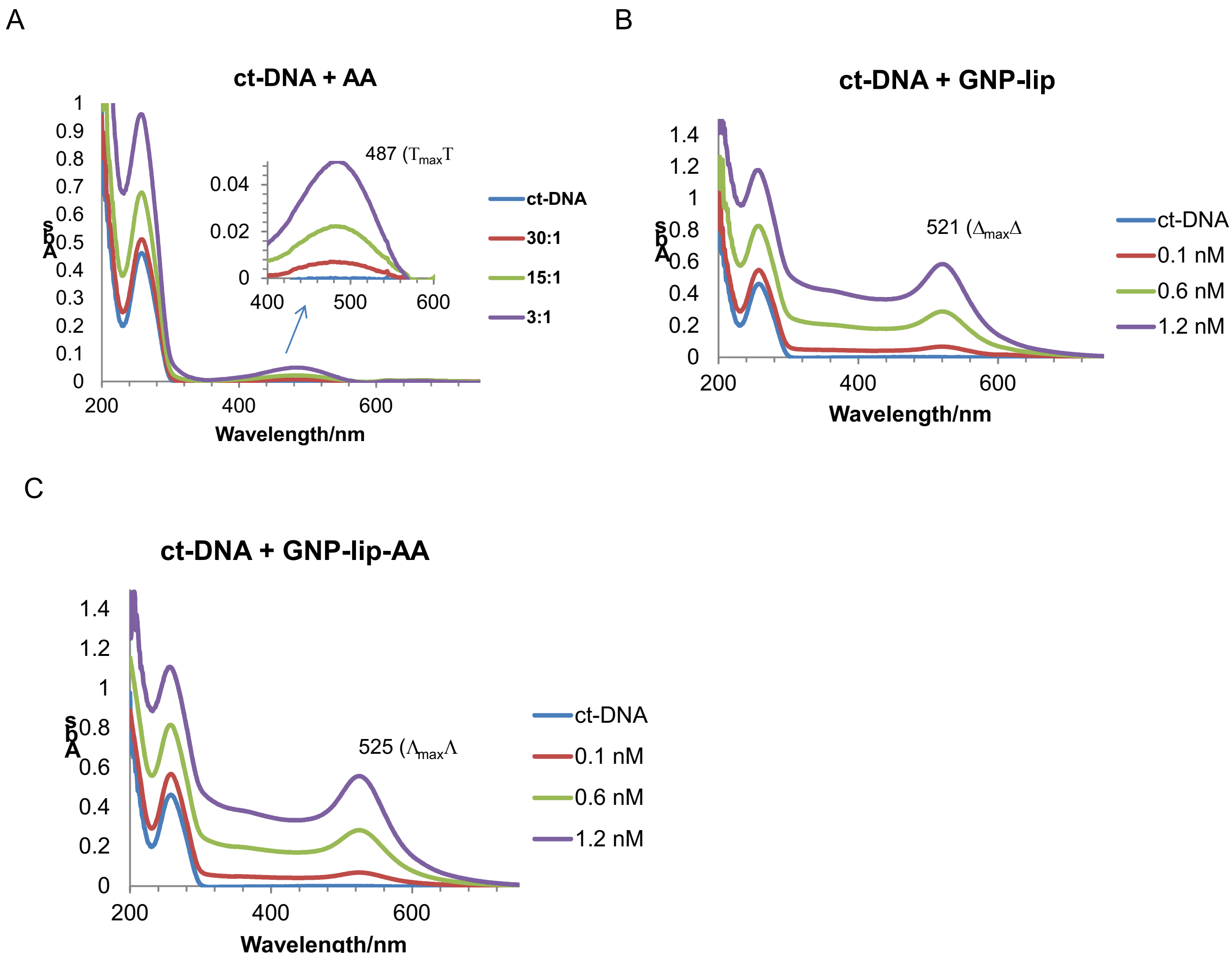

Figure S45. UV-Vis spectra of ct-DNA (75 µM in 10 mM NaCl and 1 mM Tris-HCl, pH 7.5) in the presence of increasing concentrations of A) **AA** (DNA:**AA** ratio in the legend) B) **GNP-lip** and C) **GNP-lip-AA**. Shift of λ_{max} of SPR band in the presence of DNA were not observed

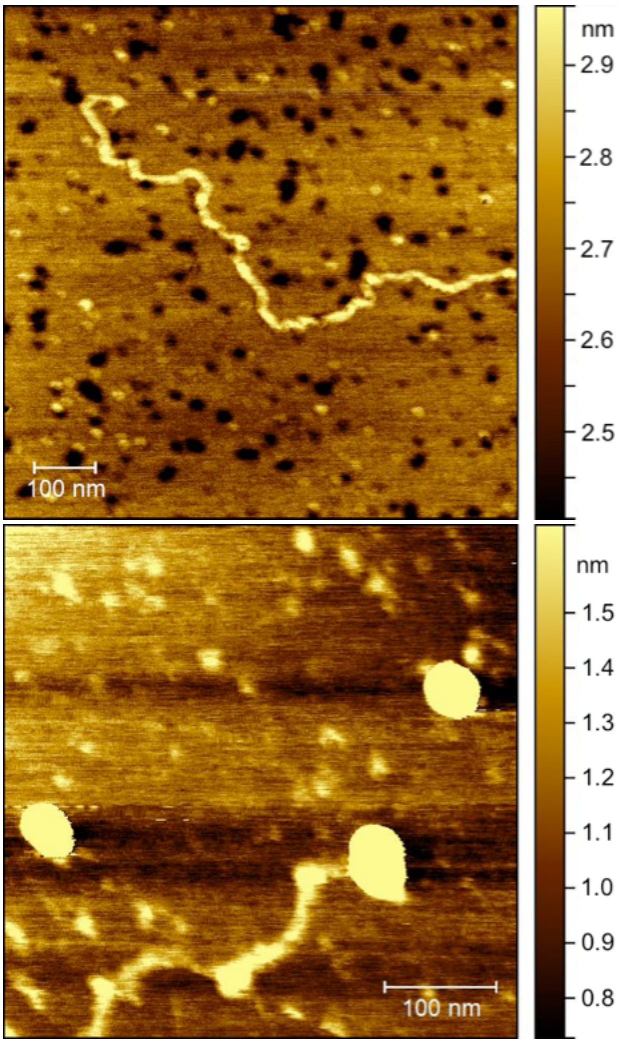

Figure S46. AFM images of linearised pBR322 plasmid DNA (top) alone and (bottom) with GNP-lip-AA.

Table S1 Comparative statistical Analysis (p-values) of IC50 data

A2780

	GNP-lip-PtII	GNP-lip-PtIVsuc	GNP-lip-AA	GNP-lip-AA-PtII	GNP-lip-AA-PtIVsuc
cisplatin	0.81			>0.99	
cisPtIVsuc-Cl		<0.05			<0.001
GNP-lip	<0.0001	<0.001	<0.0001	<0.0001	<0.0001
GNP-lip-PtII				0.86	
GNP-lip-PtIVsuc					0.49
GNP-lip-AA				0.99	>0.99

A549

	GNP-lip-PtII	GNP-lip-PtIVsuc	GNP-lip-AA	GNP-lip-AA-PtII	GNP-lip-AA-PtIVsuc
cisplatin	0.051			0.92	
cisPtIVsuc-Cl		<0.001			<0.0001
GNP-lip	<0.001	<0.0001	<0.0001	<0.0001	<0.0001
GNP-lip-PtII				<0.01	
GNP-lip-PtIVsuc					0.56
GNP-lip-AA				>0.99	>0.99

Table S2: Polydispersity values from DLS measurments

	PDI Values
GNP-lip	0.421
GNP-lip-PtII	0.461
GNP-lip-PtIV	0.444
GNP-lip-AA	0.273
GNP-lip-AA-PtII	0.402
GNP-lip-AA-PtIV	0.441
